# Salt supplementation-induced metabolic reprogramming in *Streptomyces coelicolor*

**DOI:** 10.1128/msystems.01718-25

**Published:** 2026-03-03

**Authors:** Hiroshi Otani, Katherine Louie, Meghana Faltane, Marie Lynde, Benjamin Bowen, Nigel J. Mouncey

**Affiliations:** 1 DOE Joint Genome Institute, Lawrence Berkeley National Laboratory 1666, Berkeley, California, USA; 2 Environmental Genomics and Systems Biology Division, Lawrence Berkeley National Laboratory 1666, Berkeley, California, USA; Universiteit Leiden, Leiden, Netherlands

**Keywords:** secondary metabolism, *Streptomyces*, metabolic regulation, stress response

## Abstract

Members of the genus *Streptomyces* are major producers of a wide variety of secondary metabolites that serve as bioactive compounds. Many secondary metabolites are produced in response to environmental signals such as biotic and abiotic stresses. In this study, we identified salt supplementation as one of the stimuli activating secondary metabolism in the model *Streptomyces* species, *Streptomyces coelicolor*. Comparative metabolomics revealed overproduction of several known secondary metabolites, most notably undecylprodigiosin and coelimycin P1, in addition to their biosynthetic intermediates and derivatives, as well as many unknown metabolites. Transcriptomic analysis revealed activation of diverse biological processes including cation uptake, compatible solute production, and the phosphate limitation stress response through conserved and species-specific mechanisms, presumably to overcome the increased salinity. This response leads to activation of a variety of regulatory and metabolic pathways required for production of secondary metabolites including activation of conserved metabolic pathways for energy and substrate supply and species-specific secondary metabolite biosynthetic gene clusters. Furthermore, several promoter sequences contributing to upregulation of secondary metabolism induced by salt supplementation were identified. Overall, our data show how *S. coelicolor* copes with the increased salinity and tailors the cellular metabolism toward secondary metabolism in a conserved and species-specific manner.

**IMPORTANCE:**

Precise control of cellular metabolism is critical to ensure directing cellular resources toward metabolic pathways required for the environment. Many *Streptomyces* species activate production of secondary metabolites upon exposure to environmental stimuli. This study reveals dynamic reprogramming of cellular metabolism in *Streptomyces coelicolor* under increased salinity, which induces production of various secondary metabolites. Notably, this model biological system redirects cellular resources toward various metabolic pathways required for proper activation of secondary metabolite biosynthesis, including precursor and energy supply and posttranslational modification of biosynthetic enzymes. Interestingly, some pathways are activated by phosphate limitation stress, presumably caused as a result of increased salinity. Certain aspects of this metabolic reprogramming are likely common in many *Streptomyces* species and may be controlled by rather complex regulatory pathways. Overall, this study unveils how *Streptomyces* species tailor the cellular metabolism toward secondary metabolism and paves the way for understanding metabolic regulation.

## INTRODUCTION

Members of genus *Streptomyces* are a group of bacteria belonging to Actinomycetes that undergo a complex filamentous lifestyle (1). They are also known for producing a wide variety of chemical compounds as secondary metabolites. These secondary metabolites exhibit diverse activities that are beneficial to the society, including antibiotics and agrochemicals (2). Although these metabolites are not necessarily essential for proliferation of the producing organisms, they still play pivotal roles in their environments, such as host protection (3). Most of these metabolites belong to certain metabolite classes such as polyketides and nonribosomal peptides based on their biosynthetic logic (4). Often, proteins involved in the production of a given secondary metabolite such as biosynthetic enzymes, transcription factors, transporters, and resistance proteins are encoded in the same genomic region, forming a biosynthetic gene cluster (BGC) (5). A *Streptomyces* species usually possesses ca. 30 BGCs, each producing a distinctive secondary metabolite (6). These secondary metabolite BGCs are diverse and often species- or strain-specific.

Despite the large number of BGCs each *Streptomyces* harbors, only a small number of secondary metabolites are produced under typical laboratory growth conditions. This is partly because the expression of many BGCs is tightly controlled, which enables conditional production of secondary metabolites. Various BGCs are controlled at the transcriptional level by transcription factors encoded within the same BGC (cluster-situated regulators). Examples include StrR of the streptomycin BGC in *Streptomyces griseus* and RedD of the undecylprodigiosin (*red*) BGC in *Streptomyces coelicolor* (7, 8). Many BGCs are also controlled by global regulators including AdpA and Lsr2 (9, 10). Some biosynthetic enzymes require activation by posttranslational modification. For example, polyketide synthases (PKSs) and nonribosomal peptide synthetases (NRPSs) are activated by incorporation of the phosphopantetheinyl group of coenzyme A into their acyl or peptidyl carrier domains, which is catalyzed by phosphopantetheinyl transferases (PPTases) (11). Lastly, proper substrates and co-factors need to be supplied for biosynthetic reactions. As a result, the vast majority of BGCs may be properly expressed and the encoded biosynthetic pathways activated only under specific environments. Various studies have attempted to identify growth conditions or environmental stimuli that activate otherwise silent BGCs in *Streptomyces* species (12, 13). These conditions include adding chemical compounds to the culture medium, co-cultivation with other microbes, and exposure to stress. While it is thought that production of bioactive compounds is stimulated under such environments to protect producing organisms and their hosts, how these altered environments change the physiology of the *Streptomyces* cells and enable production of secondary metabolites is largely unknown.

The model *Streptomyces* species, *S. coelicolor*, produces pigmented polyketides, namely, undecylprodigiosin, actinorhodin, and coelimycin. The blue-colored polyketide, actinorhodin, is synthesized by type II PKS encoded in the *act* BGC (14). Various analogs of actinorhodin have been discovered, including the extracellular form, γ-actinorhodin (15). Undecylprodigiosin is one of the major products of the biosynthetic pathway encoded in the *red* BGC (16). This red-colored polyketide is further circularized into streptorubin B (17). The cryptic polyketide (*cpk*) BGC directs biosynthesis of coelimycin A, although this relatively unstable compound is derived non-enzymatically into yellow-colored compounds, coelimycin P1 and P2 (18). Production of these polyketides is controlled by multiple regulatory pathways conforming complex regulatory networks (19, 20). In addition to these polyketides, peptide metabolites including calcium-dependent antibiotic (nonribosomal peptide) and siderophores, desferrioxamine E and coelichelin, have been discovered. Like many secondary metabolites, these compounds are usually produced under specific growth conditions. The changes in color by production of these pigmented metabolites were used to screen for growth conditions that induce secondary metabolism (21). However, how the production of these metabolites is enabled is still unknown. In this study, we tested the production of pigmented metabolites under various conditions and identified increased salinity as a major stimulus for secondary metabolism. Subsequently, we conducted comparative metabolomics, transcriptomics, and transcriptional start site identification to understand the metabolic and regulatory changes affected by salt supplementation. This study reveals the physiological changes that tailor the cellular metabolism toward production of secondary metabolites and paves a way for better understanding how such industrially important biological systems control bioactive compound production.

## MATERIALS AND METHODS

### Microbial cultivation

*S. coelicolor* M145 (SCP1–; SCP2–) was cultivated in 50 mL ISP-2 medium (0.4% yeast extract, 1% malt extract, and 0.4% dextrose; pH 7.2) in 250 mL Erlenmeyer flasks containing a steel spring at 28°C for 3 days. The cells were collected by centrifugation at 3,000 × *g*, washed with 10 mL sterilized water, and then resuspended in 50 mL sterilized water. To 40 mL of 1.25× ISP-2 medium, 5 mL of the cell suspension and 5 mL of 1 M sodium chloride or sterilized water were added. This cell suspension was collected as a preculture sample for RNA-seq. The cells were cultivated at 28°C for up to 96 h in 250 mL Erlenmeyer flasks containing a steel spring. At 0.5 h, 24 h, 48 h, 72 h, and 96 h, 1 mL of the culture was collected, and the cell pellet and culture supernatant were separated by centrifugation at 6,000 × *g*. The cell pellets were used to measure wet cell weights, resuspended in RNA*later*-ICE (Invitrogen), and stored at –80°C for transcriptomics until use. The culture supernatants were used to measure absorption spectra and phosphate concentrations and were stored at –20°C for metabolomics until use.

*Streptomyces griseofucsus* DSM 40191, *Streptomyces venezuelae* NRRL B-65442, and *Streptomyces rimosus* ATCC 10970 were cultivated in 50 mL ISP-2 medium in 250 mL Erlenmeyer flasks containing a steel spring at 28°C for 24 h. The cells were collected by centrifugation at 3,000 × *g*, washed with 10 mL sterilized water, and then resuspended in 50 mL sterilized water. To 40 mL of 1.25× ISP-2 medium, 5 mL of the cell suspension and 5 mL of 1 M sodium chloride or sterilized water were added. The cells were cultivated at 28°C for up to 72 h in 250 mL Erlenmeyer flasks containing a steel spring.

All the experiments were conducted three times using biologically independent replicates and independently prepared media.

### Metabolite detection, extraction, and metabolomics

The culture supernatant samples collected at 0.5 h, 48 h, and 96 h were used for spectrophotometric and metabolomics analyses. The absorbance of the culture supernatant was measured using BioTek Synergy H1 Microplate Reader (Agilent). Unused ISP-2 media were used as blanks. Metabolomics was conducted by liquid chromatography-mass spectrometry (LC-MS). The metabolite extraction was conducted using the VANTAGE Liquid Handling System (Hamilton). To 500 µL of the culture supernatant, an equal volume of ethyl acetate was added. This was mixed by pipetting 10 times, separated by centrifugation using a HiG 3 centrifuge (BioNex) at 3,000 × *g*, and the upper phase was collected. This was repeated an additional time, and the upper phases from the two extraction steps were combined. Ethyl acetate extracts were dried by using the SpeedVac vacuum concentrator (Savant), and the pellets were dissolved in 100 µL LC-MS-grade methanol containing 1 µg/mL 2-amino-3-bromo-5-methylbenzoic acid (ABMBA). The extracted samples were analyzed by LC-MS/MS using Thermo Scientific Orbitrap Exploris 120 (Thermo Fisher Scientific) and ZORBAX Eclipse Plus C18 (Agilent) following the procedure described in 22. All the biologically independent triplicate samples were used for this metabolomics analysis including the statistical analysis described below.

In order to determine whether the salt supplementation affects the ethyl acetate extraction efficiency, 500 µL ISP-2 medium supplemented with 100 mM sodium chloride or water was spiked in with 1 µg/mL of 10 known secondary metabolites, undecylprodigiosin, actinomycin D, rifampicin, thiostrepton, chloramphenicol, erythromycin, anhydrotetracycline, lincomycin, apramycin, and ivermectin, and, then, extracted with 500 µL ethyl acetate twice. These extracts were dried, dissolved in methanol containing ABMBA, and analyzed by LC-MS using the same procedure. Only two replicates were used for this extraction control experiment.

MZmine (v3.7.2) was used to generate a list of LC-MS features, to filter the features no more abundant by 3 times than water samples, and to remove isotopes. Only features detected with a retention time between 0.5 min and 8 min were used. The most intense fragmentation spectrum for each feature was uploaded to GNPS2 to determine a putative identity of the feature and structural similarity between features (23).

To identify metabolites significantly overproduced or depleted, we applied a statistical workflow. First, LC-MS features with the MS signal below 10,000,000 from all the samples were removed. Subsequently, all the MS signals were incremented by 10,000. The mean values of the MS signal from replicates were compared between data points, and the LC-MS features of which mean value did not change at least by 10-fold were removed. One-way ANOVA was conducted using the log_10_-transformed values, and only the LC-MS features with a false discovery rate-adjusted *P* value smaller than 0.05 were retained. Closer variances between sample groups, which are a requirement for ANOVA, following log_10_-transformation were verified by Levene’s test (Fig. S18). Subsequently, two-way ANOVA was conducted using the log_10_-transformed values, and false discovery rate-adjusted *P* values were calculated for two salinity conditions, three time points, and their interaction. Only the LC-MS features with the false discovery rate-adjusted *P* value for the interaction term smaller than 0.05 and of which the MS signal level changed by at least 100 times between the salinity conditions and the time points were considered significant. All the ANOVA analyses were conducted using the Python package, statsmodels.

### RNA extraction and RNA-seq

Total RNA was extracted from the cell pellets collected from the preculture samples, at 0.5 h, at 24 h, and at 48 h using the RNeasy mini kit (Qiagen). The RNA samples were treated by TURBO DNase (Invitrogen) to remove genomic DNA and cleaned up by the RNeasy mini kit. The integrities of the RNA samples were determined by using the Bioanalyzer 6000 nano kit (Agilent), and their concentrations were measured by using the Qubit RNA broad range assay kit (Invitrogen). Plate-based RNA sample prep was performed on the Sciclone NGS robotic liquid handling system (PerkinElmer) using the FastSelect 5S/16S/23S for bacterial rRNA depletion kit (Qiagen) with RNA blocking oligo technology to block and remove rRNA from 100 ng of the total RNA input. An Illumina sequencing library was then created from the fragmented and rRNA-depleted RNA using the TruSeq Stranded Total RNA HT sample prep kit (Illumina) with 10 cycles of PCR for library amplification. The prepared libraries were quantified using the next-generation sequencing library qPCR kit (KAPA Biosystems) and run on a LightCycler 480 real-time PCR instrument (Roche). Sequencing of the flow cell was performed on the Illumina NovaSeq X Plus sequencer using NovaSeq X Plus reagent kits and flow cell, following a 2 × 150 nt indexed run recipe.

Sequencing reads were quality controlled using BBTools (24). Raw reads were evaluated for artifact sequences by K-mer matching (kmer = 25), allowing 1 mismatch, and the detected artifact was trimmed from the 3′ end of the reads. RNA spike-in reads, PhiX reads, and reads containing any Ns were removed. Quality trimming was performed using the phred trimming method set at Q6. Finally, following trimming, reads under the length threshold were removed (minimum length 25 nt or 1/3 of the original read length—whichever is longer). Trimmed reads were aligned to the *S. coelicolor* A3(2) chromosome sequences using HISAT2 with the “no-spliced-alignment” option and the “maxins” option of 1,000 (25). The number of fragments overlapping each gene was counted using *featureCounts* (26). Differential expression was analyzed using the R package *DESeq2* (27). Log_2_-transformed fold change relative to the preculture samples was used to analyze the time- and salinity-dependent expression levels. The log_2_-transformed fold change was set to 0 if the false-adjusted *P* value was ≥0.05. All the biologically independent triplicate samples were used for this transcriptomics analysis.

### Cappable-seq

The RNA samples collected at 24 h and 48 h were used for Cappable-seq (28). RNA samples were enriched in 5′ ends of the primary transcripts using the procedure provided by New England Biolabs. Briefly, 5′ ends of 2 µg RNA were labeled with desthiobiotin-GTP (DTB-GTB; NEB) by Vaccine Capping Enzyme (NEB) and fragmented by incubation at 70°C for 2 min, followed by dephosphorylation by ATP-free T4 polynucleotide kinase (Thermo Scientific). The labeled transcripts were enriched by using hydrophilic streptavidin magnetic beads (NEB) twice, and the 3′ adapter (TGGAATTCTCGGGTGCCAAGG) was ligated. The enriched transcripts were decapped by RppH (NEB) in a Thermopol Buffer (NEB), and the 5′ adapter (GUUCAGAGUUCUACAGUCCGACGAUC) was ligated. cDNA was synthesized by using SuperScript IV (Invitrogen) with the RT primer (GCCTTGGCACCCGAGAATTCCA). Indexing primers from the TruSeq Small RNA Library Prep Kit (Illumina) and KAPA HiFi HotStart Ready Mix (Roche) were used to amplify the cDNA. The amplified libraries were size-selected by using AMPure XP Beads (Beckman Coulter) to obtain DNA fragments primarily between 200 bp and 500 bp (average size of 350–400 bp). Bioanalyzer High Sensitivity DNA electrophoresis (Agilent) was conducted to determine the DNA size distribution of the libraries. The quantities of the libraries were determined by using the KAPA Library Quantification Kit on the LightCycler 480 instrument (Roche). These libraries were pooled and sequenced by the NovaSeq X Plus sequencer using NovaSeq ZP v1.5 reagent kits (Illumina) to generate 2 × 151 nt reads.

Raw reads were scanned from 3′ to 5′, those with a quality score value below 20 were trimmed, and reads consisting of fewer than 35 nucleotides were discarded using BBDuk (24). Trimmed reads were aligned to the *S. coelicolor* A3(2) chromosome sequences using HISAT2 with the “no-spliced-alignment” option and the “maxins” option of 1,000 (25). The number of 5′ ends of the aligned read pair was calculated at each genomic position to find potential transcription start sites (TSSs). The potential TSSs were clustered if they were located within 5 nt, and the genomic position of the 5′ end with the greatest count was used. Subsequently, these clustered TSSs were compared between replicates and further clustered if they were within 5 nt. Only the TSSs detected in all the replicates were used for further analysis. TSSs determined under 4 different conditions (2 time points under 2 different salinity conditions) were compared, and their locations were clustered if they were located within 5 nt. MEME version 5.5.8 was used to identify a consensus promoter motif (29). All the biologically independent triplicate samples were used for this analysis.

### Functional assignment

InterProScan version 5.71, eggNOG-mapper version 2.1.12, and KofamScan version 1.3.0 were used to assign InterPro accession numbers, evolutionary genealogy of genes: nonsupervised orthologous groups (eggNOG), and KEGG ortholog (KO) numbers to each protein sequence, respectively, using the predefined thresholds (30–34). Accession numbers used to identify proteins involved in potassium uptake are listed in Table S1. The *Streptomyces* pangenome data set was used to calculate the conservation of each protein (https://smc.jgi.doe.gov/projects/) (6). The secondary metabolite BGCs were predicted by antiSMASH version 7.1.0 (35). Information about the characterized BGCs was retrieved from the Minimum Information about a Biosynthetic Gene (MIBiG) cluster database version 4.0 (36). The consensus genome-scale metabolic model of *S. coelicolor* was retrieved from the GitHub repository (https://github.com/SysBioChalmers/Sco-GEM) (37). Fisher’s exact test was conducted for the gene set enrichment analysis.

### Phosphate assays

The Phosphate Assay Kit (Sigma-Aldrich) was used to measure the phosphate concentrations in the culture broth. To 10 µL of malachite green reagent, 5 µL of the culture supernatant diluted by 10 times with pure water was added. The mixture was incubated for 30 min at room temperature, and the absorbance at 620 nm was measured. The phosphate standard included in the kit was used to generate a standard curve for calculation. All the biologically independent triplicate samples were used for this analysis.

## RESULTS

### Salt supplementation induces secondary metabolite production

Many bacteria, including the model organism *S. coelicolor* A3(2), produce different secondary metabolites in response to external stimuli including abiotic stresses such as temperature shift and antibiotics and presence of other organisms (21, 38, 39). However, it is difficult to predict such stimuli. In order to identify environments that promote secondary metabolite production, we cultivated *S. coelicolor* M145 (plasmid-free strain) in ISP-2 liquid medium under 32 different cultivation conditions (Table S2; Fig. S1) and sampled at different time points. Although ISP-2 medium is known to promote production of pigmented secondary metabolites in *S. coelicolor* including undecylprodigiosin, it requires prolonged cultivation (40). We screened for conditions that enhance pigmentation of the culture medium within 4 days. The 32 cultivation conditions tested include increased osmolarity, low or high temperature, addition of an antibiotic or a metal, oxidative stress, and co-cultivation. Of these conditions, the salt-supplemented condition (100 mM sodium chloride added) most substantially induced red pigmentation of the culture broth, indicative of undecylprodigiosin production (41, 42). This concentration of 100 mM, which is relatively lower than those used in the previous studies, was chosen to ensure similar growth phases between conditions at the same time points because the previous studies on salt-supplemented conditions using higher concentrations (500 mM–1,000 mM) showed delayed growth or cellular differentiation (43, 44). Nevertheless, this concentration was sufficient to change the extent of pigmentation. The spectrophotometric analysis of the culture supernatants showed the salt-supplemented condition increased absorption across a range of wavelengths including 540 nm, the absorbance maximum of undecylprodigiosin (Fig. S2) (45). These observations suggest production of various metabolites by salt supplementation including undecylprodigiosin.

### Identification of secondary metabolites overproduced by increased salinity

**Fig 1:**
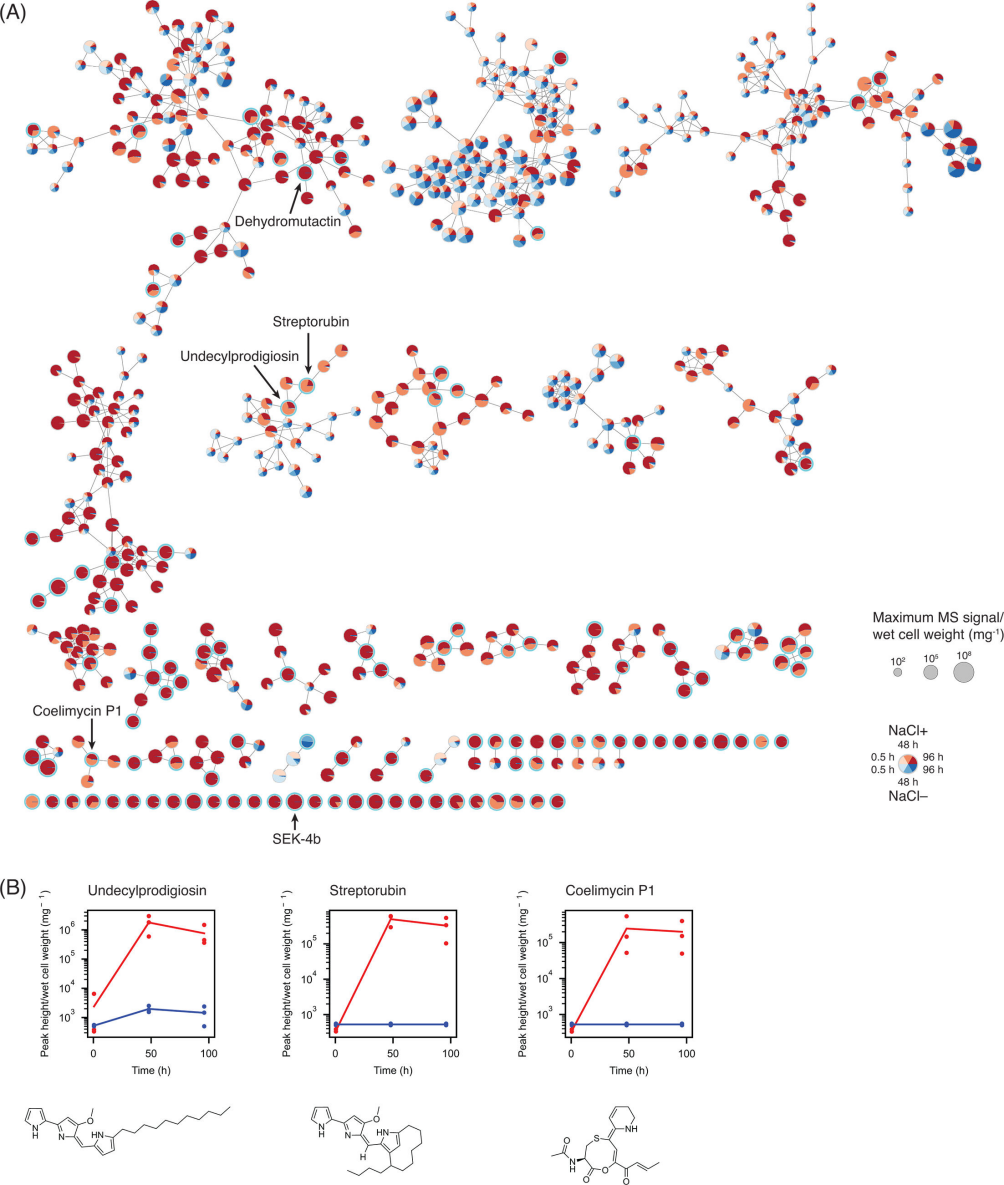
Metabolomics analysis of the culture supernatant under the salt-supplemented condition (*n* = 3 independent biological experiments). (**A**) Molecular network analysis of the metabolites detected in the positive ionization mode. The nodes with a cyan outer circle indicate metabolites significantly differentially produced or depleted between two salinity conditions. Only clusters containing at least one metabolite significantly produced or depleted are shown. The node size indicates the maximum mass spectrometry (MS) signal quantity per wet cell weight. (**B**) Quantities of undecylprodigiosin, streptorubin, and coelimycin P1 detected in the positive ionization mode. Lines connect average values from three independent biological experiments. Values are normalized by wet cell weight. Red line and dots: salt-supplemented condition. Blue line and dots: no salt addition.

Because of the increased production of metabolites, including undecylprodigiosin, by salt supplementation, we conducted a comparative metabolomic analysis by LC-MS to identify secondary metabolites overproduced by increased salinity. The culture supernatants at three time points (0.5 h, 48 h, and 96 h) were extracted by ethyl acetate and analyzed by LC-MS in positive and negative ionization modes. Note that we also conducted the same extraction and LC-MS analyses with the medium supplemented with or without 100 mM sodium chloride and spiked in with 10 known secondary metabolites produced by various actinomycetes and their derivatives. Of these 10 compounds, 8 compounds were detected by LC-MS analysis, and the signal levels of these compounds did not significantly change by salt supplementation, indicating no detectable effect of salt supplementation on the extraction efficiency (Fig. S3). Following the LC-MS analysis of the culture supernatant samples, the untargeted analysis and MS spectral search by GNPS revealed production of several known secondary metabolites (23). These included germicidins (46), although their production level was not detectably affected by the increased salinity. The subsequent statistical analysis revealed that a total of 161 and 123 LC-MS features were determined to be differentially produced or depleted in positive and negative ionization modes, respectively (Fig. 1A; Fig. S4, Tables S3 and S4). Consistent with the absorbance spectrum data, undecylprodigiosin was more abundantly produced under the high-salinity condition together with streptorubin B, a cyclic derivative of undecylprodigiosin (17) (Fig. 1B). Additionally, coelimycin P1 was more actively produced. Furthermore, there are several polycyclic compounds that more abundantly accumulated under the salt-supplemented condition. One of them, hesperetin, is a flavonoid produced by type III PKS. Its production was previously observed in *Streptomyces albidoflavus,* although its corresponding type III PKS has not been identified in any *Streptomyces* species (47). Interestingly, known shunt products of actinorhodin biosynthesis and their derivatives such as SEK-4b and dehydromutactin were overproduced under the increased salinity condition (48–50). We, therefore, conducted a targeted analysis of the LC-MS data to determine the production of actinorhodin. There were several peaks whose mass matched with that of actinorhodin and its derivatives including the extracellular form of actinorhodin and the final product of the actinorhodin biosynthesis, γ-actinorhodin. Consistent with the accumulation of the shunt products of actinorhodin biosynthesis, the MS signals of these putative actinorhodin features were higher under the increased salinity condition, suggesting enhanced actinorhodin biosynthesis (Fig. S5). Overall, our metabolomics data indicate active production of several secondary metabolites by increased salinity.

### Salt supplementation-induced differential expression

**Fig 2:**
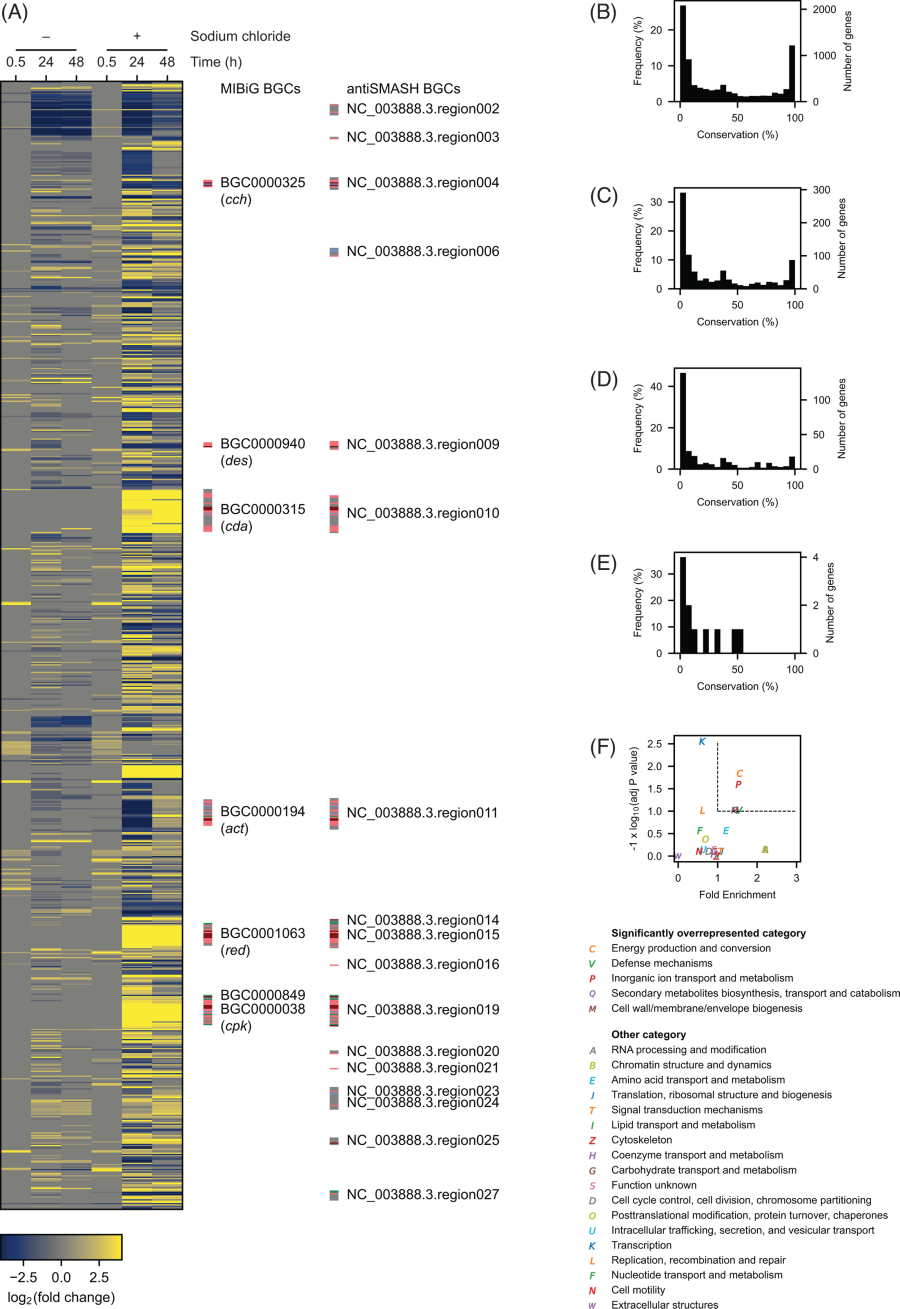
Transcriptomics analysis in response to the increased salinity. (**A**) Heatmap of differentially expressed genes (*n* = 3 independent biological experiments). The relative fold change to the preculture condition was used. Secondary metabolite biosynthetic gene clusters (BGCs) available in Minimum Information about a Biosynthetic Gene (MIBiG) and predicted by antiSMASH are shown on the right side. The color codes indicate the functions (dark red: core biosynthetic genes, light red: additional biosynthetic gene, light blue: transport-related gene, green: regulatory gene, gray: other genes). The number of genes in each conservation bin from (**B**) all the genes, (**C**) differentially expressed genes, (**D**) genes more highly expressed by salt supplementation, and (**E**) genes more highly expressed without salt supplementation. (**F**) Functional enrichment analysis of differentially expressed genes. Dashed lines indicate false discovery rate-adjusted *P* value of 0.1 (horizontal line) and fold enrichment of 1 (vertical line).

We conducted a transcriptomic analysis to better understand the physiological changes caused by increased salinity. Because transcription of some metabolic genes may be temporal and may not match with the timing of metabolite accumulation, transcriptomes at 3 time points, which include an earlier time point than those used for the metabolomics analysis (0.5 h, 24 h, and 48 h), were analyzed by RNA-seq (51, 52). The later time points used in the metabolomics are excluded due to the poor qualities of the RNA sample, likely caused by a higher proportion of dead cells. The preculture sample that was inoculated into the main culture was used as the reference condition (time 0). The correlation analyses of the sequence fragment count per gene showed clustering of and high Pearson correlation coefficients between replicates, suggesting a high level of reproducibility between replicates (Fig. S6). The log_2_-transformed fold change relative to the reference condition was used for the further analyses. The log_2_-transformed fold change was set to 0 (no change) if the false discovery rate-adjusted *P* value was equal to or greater than 0.05. A gene was considered differentially expressed if the fold change between the highest and lowest expression levels among the 7 conditions (3 time points with or without sodium chloride plus the reference condition) was 5-fold or greater. A total of 894 genes satisfy this criterion and are considered differentially expressed (Fig. 2A; Table S5). Conservation of these differentially expressed genes was analyzed using our *Streptomyces* pangenome data set (6). Compared to all the genes the *S. coelicolor* genome harbors, differentially expressed genes were conserved to a lesser extent, suggesting that salt supplementation affects species- or strain-specific biological processes in addition to conserved pathways (Fig. 2B and C). We conducted a Fisher’s exact test to determine functional categories overrepresented in the differentially expressed genes (Fig. 2F). Using the functional categories assigned by EggNOG, genes belonging to “Energy production and conversion,” “Defense mechanisms,” “Inorganic ion transport and metabolism,” “Secondary metabolites biosynthesis, transport and catabolism,” and “Cell wall/membrane/envelope biogenesis” were overrepresented by 1.4–1.6 times with the adjusted *P* value < 0.1 (Fig. 2F). The majority of the genes in the categories with enrichment levels < 1 are likely either constitutively or hardly expressed under the conditions used in this study. For example, the category “Transcription” had the enrichment level of 0.63. *Streptomyces* genomes encode close to 1,000 transcription factors such as transcriptional activators, repressors, and sigma factors (6), and the majority of them are presumed to be involved in adaptation to specific environmental signals or cellular processes (53). For example, various transcription factors encoded by *whi* genes are involved in sporulation in several *Streptomyces* species (54–58). Since *S. coelicolor* does not form spores in liquid cultures, many *whi* genes are unlikely to be differentially expressed in liquid cultures regardless of the salt concentration.

While the majority of differentially expressed genes were primarily affected by increased salinity, there are still several genes whose expression patterns were mainly growth phase-dependent irrespective of the salt concentration (Fig. S6). For example, many genes encoding ribosomal proteins were highly expressed at 0.5 h under both conditions (Fig. S7A). Similarly, the majority of genes involved in nitrate reduction (*SCO0203–SCO0220*) for anaerobic respiration were highly expressed at the beginning of the growth (Fig. S7B) (59, 60). Additionally, *nsrR* (*SCO7427*) encoding the nitric oxide (NO) sensor transcription factor was upregulated, suggesting active conversion of nitric oxide to ammonium during the exponential phase (61). Although the expression of these genes decreased at later time points (24 h and 48 h) irrespective of the salt concentration, their expression levels were slightly higher under the salt-supplemented condition. These data suggest that cells maintain a relatively higher level of metabolic activity under the salt-supplemented condition, particularly anaerobic respiration, which may reflect possible reduced oxygen solubility in the salt-supplemented medium (60). There are several genes whose expression increased at later time points under both conditions. We speculate that some of them are involved in pathways related to adaptation to the stationary-phase environments. For example, several transcription factor genes including *lsr2* and *wblC* are upregulated (Fig. S7C). The nucleoid-associated protein, Lsr2, primarily suppresses the expression of laterally acquired genes, particularly BGCs, to, presumably, safeguard the cell physiology by avoiding inappropriate gene expression in *S. venezuelae* (10). The WhiB-like transcription factor, WblC, controls resistance to translation-targeting antibiotics. However, the functions of many of the growth phase-dependent genes are not definitively known or predicted.

**Fig 3:**
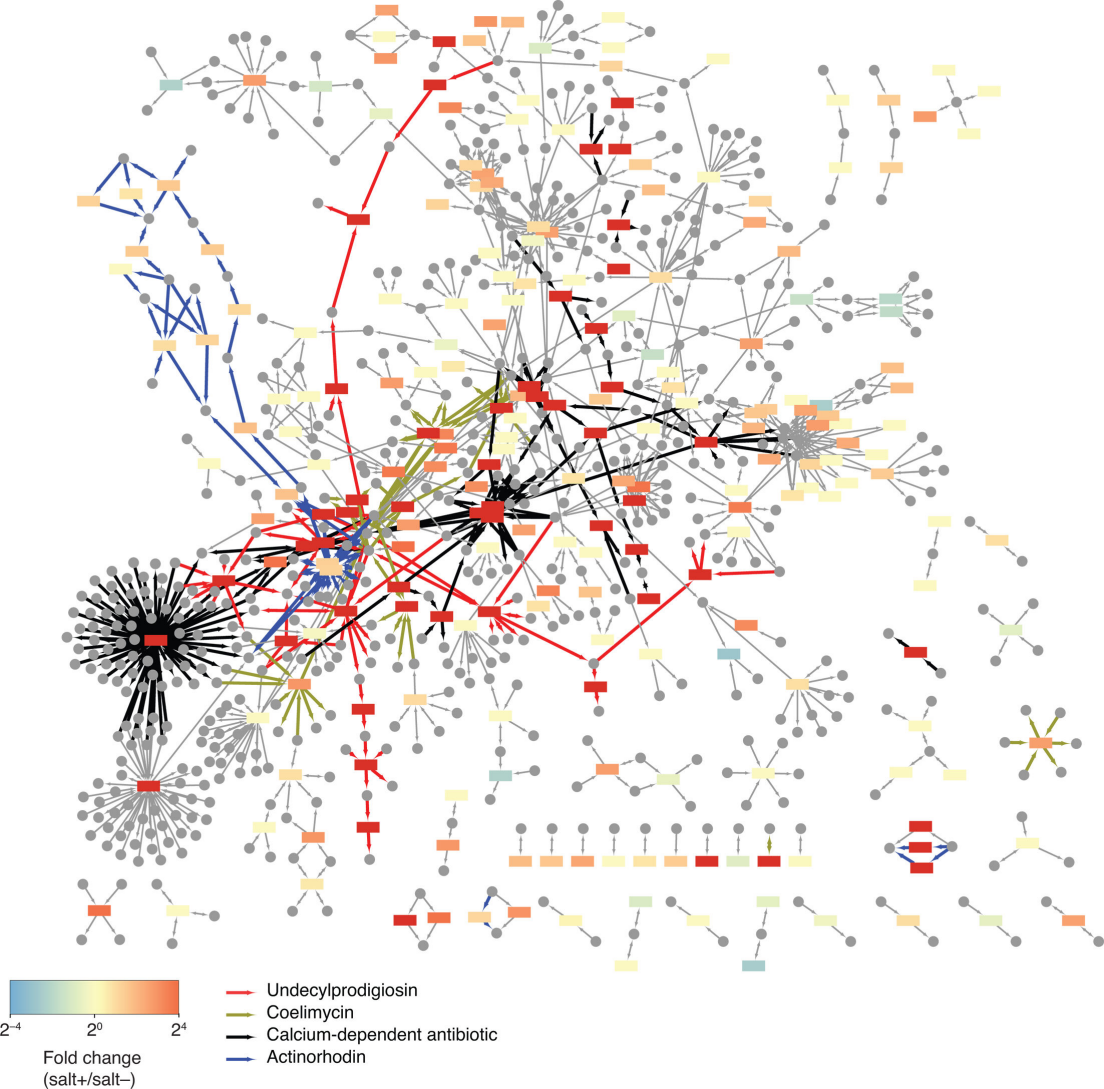
The comparative transcriptomic data overlaid on the *S. coelicolor* consensus genome-scale metabolic model. Rectangles are proteins, and circles are metabolites. Only the proteins encoded by differentially expressed genes are shown. The color of the rectangles indicates the fold change of expression (salt+/salt–). For example, the red-colored rectangles indicate genes encoding these reactions more and actively transcribed under the salt-supplemented condition, while the blue-colored rectangles indicate genes more highly expressed when salt was not supplemented. Colored arrows are enzymes catalyzing biosynthetic pathways of four secondary metabolites.

To find genes whose transcription was enhanced by increased salinity, the maximum level of transcription between 3 time points under the salt-supplemented condition was compared to the maximum level of transcription between 3 time points without salt supplementation. We considered a gene more actively transcribed if this fold change was 5 times or greater. This analysis ensures a given gene is more actively transcribed at least at 1 time point under 1 salinity condition (e.g., salt supplementation) than all the time points under the other salinity condition (e.g., no salt supplementation). Of 894 genes differentially expressed, 306 genes were determined to be more highly expressed under the high-salinity condition, while 11 genes turned out to be more actively expressed when salt was not supplemented. These genes are conserved even to a lesser degree (Fig. 2D and E). We then mapped the differentially expressed genes to the consensus genome-scale metabolic model of *S. coelicolor* (Fig. 3) (37). Of 894 differentially expressed genes, 262 genes are in this metabolic model. The salt-supplemented condition induced the expression of 107 genes in the metabolic model, while only 3 genes were highly expressed when no salt was added. In the following subsections, we focus on genes whose expression levels changed in response to the increased salinity.

#### Potassium uptake

**Fig 4:**
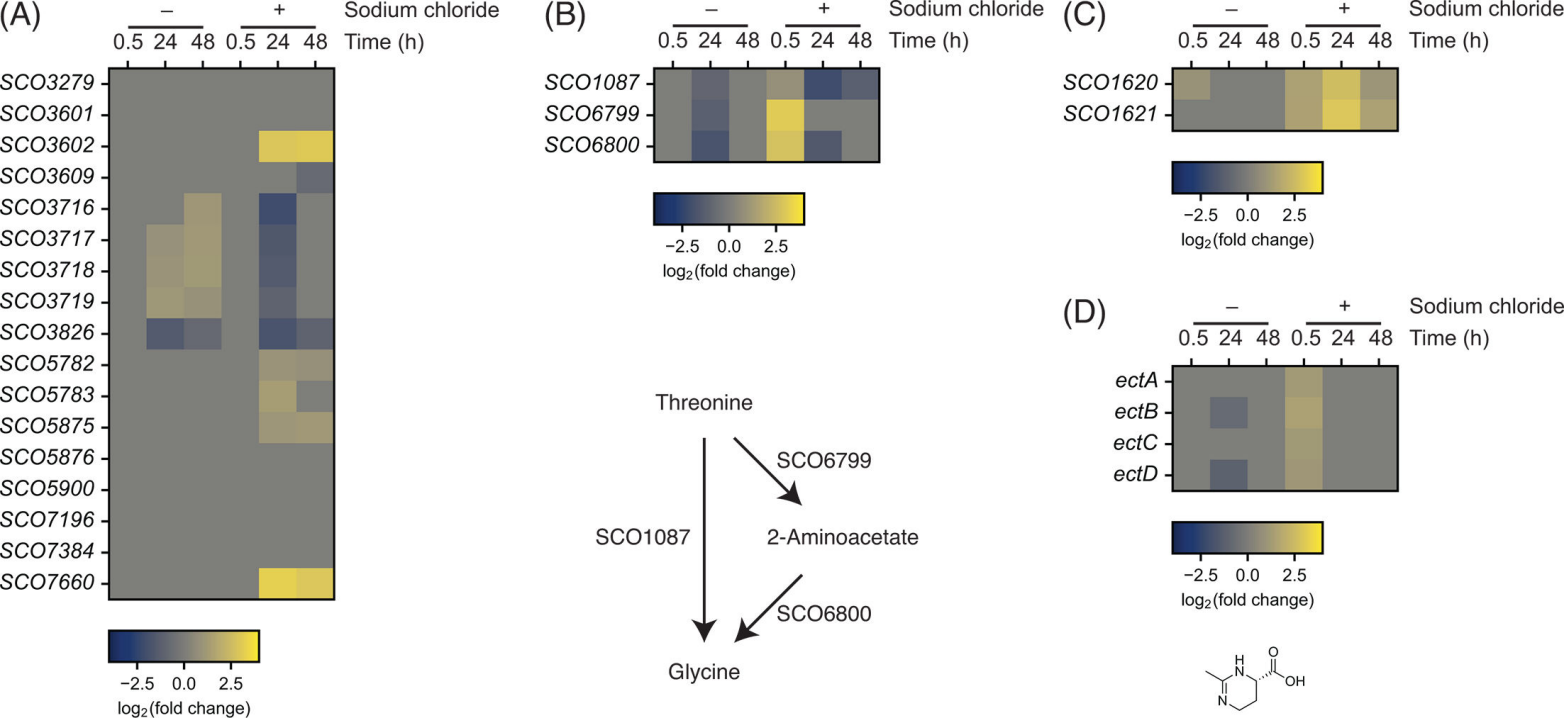
Heatmap of genes involved in (**A**) potassium/cation uptake, (**B**) threonine conversion to glycine, (**C**) glycine uptake (OpuA homologs), and (**D**) ectoine biosynthesis (*n* = 3 independent biological experiments). The color-coding indicates log_2_-transformed fold change relative to the reference condition.

Many bacteria uptake potassium cations from the environment upon exposure to high osmolarity, including salinity stress, to avoid dehydration of cells (62). Indeed, accumulation of potassium cations by increasing the salt concentration was previously observed in *Streptomyces griseus* and *Streptomyces californicus* (63). *S. coelicolor* encodes 18 proteins predicted to belong to one of the protein families involved in potassium uptake (64). Of these, *SCO7660* was the most highly upregulated gene under the salt-supplemented condition (Fig. 4A). This gene encodes a biochemically characterized cation channel, KcsA (65, 66). The prior biochemical and structural studies have shown that KcsA contains polyphosphate and poly[(*R*)−3-hydroxybutyrate] and transports preferentially divalent cations such as magnesium and calcium cations at pH >7 and potassium cations at pH <7 (67, 68). However, its physiological role has not been studied. Additionally, *SCO3602*, encoding a cation/H^+^ antiporter, was strongly upregulated. Interestingly, its neighboring genes also encode proteins belonging to one of the protein families for potassium uptake (SCO3601 and SCO3609) or contain protein domains likely involved in potassium uptake (SCO3603 containing a cation/H^+^ exchanger domain and a regulator of K^+^ conductance C-terminal domain). Of these, *SCO3603* was upregulated. Upregulation of these genes strongly suggests the uptake of cations, most likely potassium cations, into *S. coelicolor* cells in response to increased salinity. Interestingly, SCO3602 homologs are encoded on 146 genomes out of 205 *Streptomyces* species examined, while KcsA is only conserved in 7 species (6). It could be plausible that the *SCO3602* and its surrounding genes are responsible for potassium uptake in diverse *Streptomyces* species, whereas KcsA provides an additional potassium or other cation uptake system to only *S. coelicolor* and its closely related species.

Unexpectedly, the transcriptional unit encoding the orthologs of KdpFABC in *Escherichia coli*, *SCO3716–SCO3719*, was mainly downregulated. The KdpFABC complex pumps potassium cations into the cells by using ATP in *E. coli* (69, 70). This observation suggests that the KdpFABC orthologs are unlikely the primary transporter of potassium cations under the conditions used in this study.

#### Amino acid uptake and compatible solute production

In addition to potassium uptake, bacteria also import or synthesize compatible solutes such as glycine betaine upon osmotic stress (71). Indeed, a previous study showed that *S. coelicolor* accumulates small peptides containing glycine and proline in response to salinity stress (44). The transcriptome data showed upregulation of one of the pathways converting threonine to glycine via 2-aminoacetate catalyzed by SCO6799 and SCO6800 at 0.5 h upon salt supplementation (Fig. 4B). Additionally, genes encoding homologs of the glycine betaine transporter in *B. subtilis*, OpuA, were upregulated (Fig. 4C) (72). These data suggest *S. coelicolor* activates glycine accumulation to counteract the increased salinity. Interestingly, transcription of the genes involved in proline biosynthesis was not detectably affected by salt supplementation. It is unclear whether proline accumulation was also activated under the conditions used in this study.

The previous studies also showed increased accumulation of ectoine inside cells and its involvement in salt and heat stress protection as compatible solutes (44, 73). The genes in the ectoine BGC were relatively highly expressed at the earlier time point under the salt-supplemented condition (2–3 times) (Fig. 4D). Our metabolomics analysis primarily detects hydrophobic compounds from the culture supernatants and failed to detect intracellular accumulation of ectoine and its derivatives. Given the high-level conservation of this BGC in the *Streptomyces* genomes and the known function of ectoines as compatible solutes, enhancement of ectoine BGC expression could be conserved within the genus *Streptomyces* (6, 73). It is interesting to compare the ectoine quantity in response to increased salinity under the condition used in this study. Overall, our data support the possibility of accelerated metabolism for compatible solute production to counteract increased salinity.

#### Phosphate limitation stress response genes

**Fig 5:**
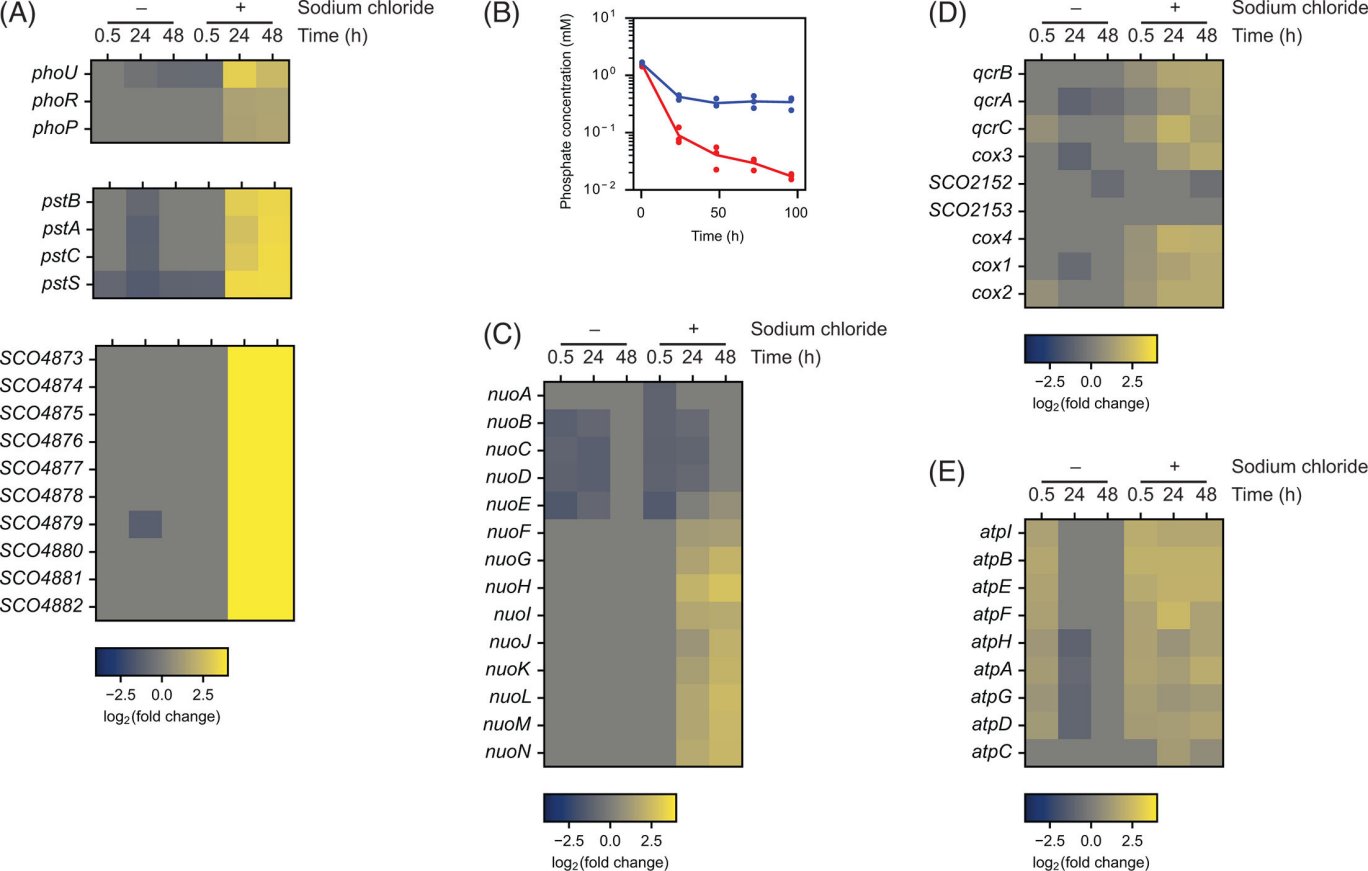
Heatmap of (**A**) the *pho* gene cluster, the *pst* gene cluster, and the teichulosonic acid-cell wall biosynthetic gene cluster, (**C**) the *nuo* genes, (**D**) the *cox* and *qcr* genes, and (**E**) the *atp* genes (*n* = 3 independent biological experiments). The color-coding indicates log_2_-transformed fold change relative to the reference condition. (**B**) Phosphate concentrations in the culture medium. Red line and dots: salt-supplemented condition. Blue line and dots: no salt addition. Lines connect average values from three independent biological experiments.

One of the COG categories highly enriched by the gene set enrichment analysis was “Inorganic ion transport and metabolism.” Many genes belonging to this category are involved in phosphate uptake and utilization. Most notably, the *pstSCAB* operon, encoding the high-affinity phosphate transport system, was highly expressed (>10 times at 48 h) (Fig. 5A). The *pstSCAB* operon is known to be directly activated by the two-component response regulator, PhoP (74). Consistent with this observation, other major target genes of PhoP such as teichulosonic acid-cell wall biosynthetic genes (*SCO4873–SCO4882*) were highly expressed by salt supplementation (74, 75). PhoP activity is controlled via phosphorylation, which is catalyzed by PhoR in response to phosphate limitation (74). Since PhoP activity is likely an indication of phosphate limitation, we measured the phosphate concentration in the culture medium (Fig. 5B). With no salt supplementation, the phosphate concentration decreased from ca. 1.6 mM to ca. 0.4 mM within 24 h and gradually decreased to ca. 0.3 mM over 96 h. The increased salinity accelerated phosphate depletion from the medium, and it decreased to <0.02 mM over 96 h. Therefore, it is plausible that accelerated depletion of phosphate induced the phosphate limitation response and activated the PhoP regulon.

We, then, wondered whether the accelerated phosphate depletion is a common phenomenon in various *Streptomyces* species. Therefore, we cultivated three different *Streptomyces* species, *Streptomyces griseofuscus*, *Streptomyces venezuelae,* and *Streptomyces rimosus*, in ISP-2 medium with or without salt supplementation and measured the phosphate concentrations over time. Interestingly, the rate of phosphate depletion did not noticeably change by salt supplementation in these cultures (Fig. S8). The phosphate concentration was almost constant in the *S. rimosus* culture throughout the growth. The undetectable depletion of phosphate in the *S. rimosus* culture was somewhat unexpected, which might indicate the use of other phosphate sources present in the medium, such as vitamins and phospholipids, or more efficient re-use or recycling of metabolites. Nevertheless, these results suggest that accelerated phosphate depletion upon salt supplementation is unlikely a common response mechanism among various *Streptomyces* species. These results also ensure that accelerated depletion of phosphate from the culture supernatant was unlikely a result of the artifact of measurement or phosphate solubility caused by increased salt concentration. A possible cause of accelerated phosphate depletion is discussed in the Discussion section.

#### Energy production

The other COG category that was highly enriched was “Energy production and conversion.” One of the genomic loci differentially expressed was the *nuo* gene cluster, encoding the NADH dehydrogenase complex I, which catalyzes the first step of oxidative phosphorylation to provide electrons to the respiratory chain (Fig. 5C). Of 14 genes from this gene cluster, 10 genes were more actively expressed under the salt-supplemented condition. Interestingly, we noticed antisense transcription of the first 6 genes, which might affect translation of these genes (Fig. S9). The antisense transcription was more active when sodium chloride was not supplemented, possibly suggesting reduced translational regulation of the *nuo* genes by salt supplementation. We wondered whether the differential expression of the *nuo* gene cluster was caused by the phosphate depletion. We, therefore, analyzed the transcriptome data from the cultures with varying phosphate concentrations (76). Many of the *nuo* genes were more highly expressed with reduced phosphate availability, including antisense transcription, suggesting phosphate limitation induced expression (Fig. S10A and B).

Additionally, genes encoding the cytochrome *c* oxidase (*cox*) and reductase (*qcr*) were more actively transcribed (Fig. 5D). Unlike the *nuo* gene cluster, these genes were not highly expressed under the phosphate limitation conditions (Fig. S10C). Finally, the F-type ATP synthase genes were actively transcribed throughout growth under the increased salinity condition, while their expression level was high only at the early time point when salt was not supplemented (Fig. 5E). F-type ATP synthase catalyzes ATP synthesis from ADP and phosphate. Similar to the *cox* and *qcr* genes, the ATP synthase genes were not differentially expressed by phosphate limitation (Fig. S10D). Overall, various genes involved in oxidative phosphorylation to produce ATP were highly expressed by increased salinity. However, the expression of only the *nuo* gene cluster is likely induced by phosphate limitation as a result of increased salinity.

#### Secondary metabolism

**Fig 6:**
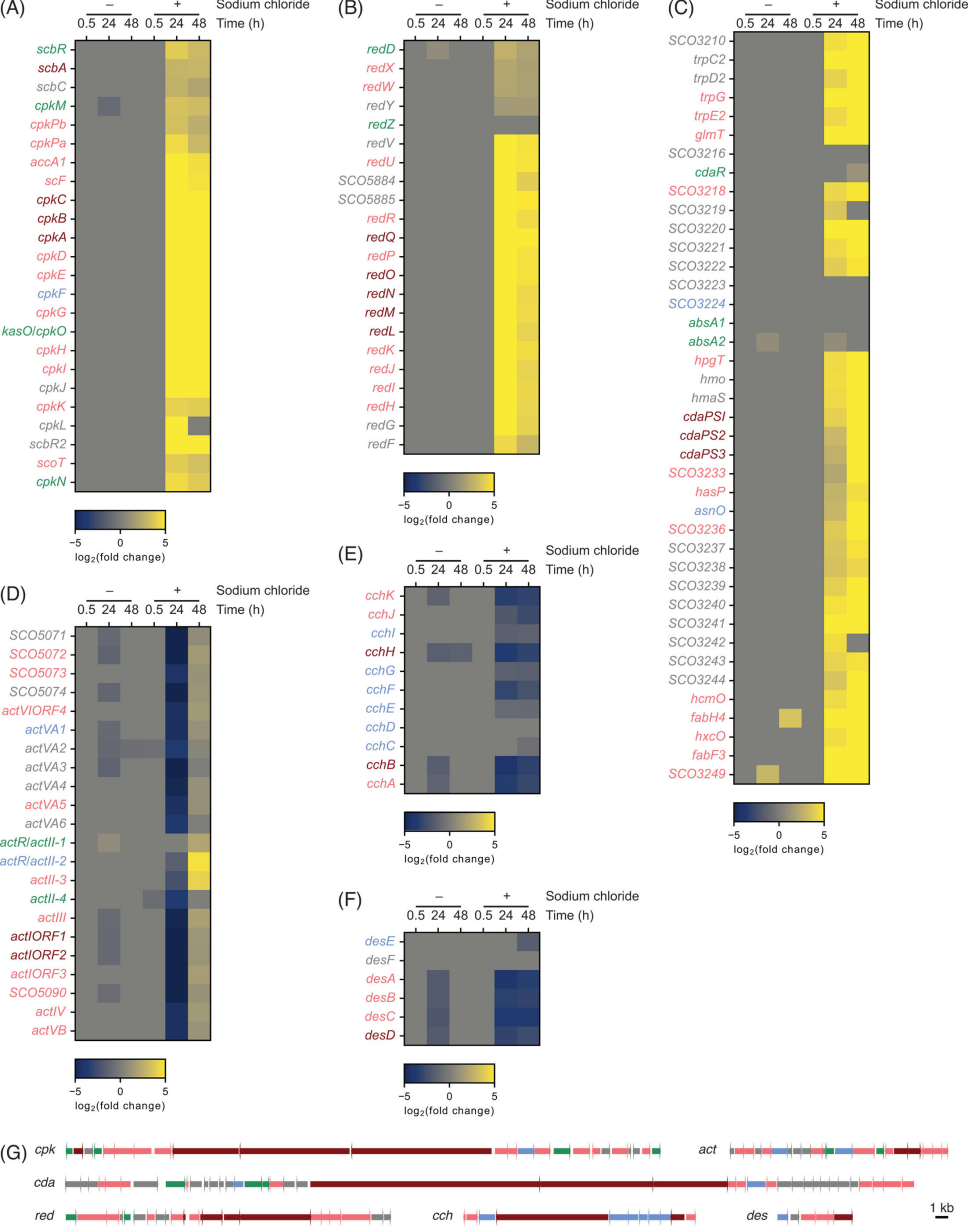
Heatmap of (**A**) the *cpk* BGC, (**B**) the *red* BGC, (**C**) the *act* BGC, (**D**) the *cda* BGC, (**E**) the *cch* BGC, and (**F**) the *des* BGC (*n* = 3 independent biological experiments). The color-coding indicates log_2_-transformed fold change relative to the reference condition. (**G**) Gene organization of the BGCs. The scale bar indicates 1 kb. The arrows from left to right correspond to genes from the top to bottom of the respective heatmap in **A–F**. Color codes of the genes indicate the functions (dark red: core biosynthetic genes, light red: additional biosynthetic gene, light blue: transport-related gene, green: regulatory gene, gray: other genes).

The transcriptomics data show differential expression of various genes involved in secondary metabolism (Fig. 2A and F, Fig. 3 and 6). Consistent with the metabolomics data, the coelimycin (*cpk*) and undecylprodigiosin (*red*) BGCs were more actively transcribed under the salt-supplemented condition (Fig. 6A and B). The *cpk* BGC also encodes an enzyme required for SCB biosynthesis (ScbA) (77). As expected, the MS signals matching the theoretical *m*/*z* values of SCB-1, SCB-2, and SCB-3 increased in response to increased salinity (Fig. S11). The other highly expressed BGC was *cda*, responsible for calcium-dependent antibiotic biosynthesis (Fig. 6C) (78). However, presumably due to its relatively high hydrophilicity, the metabolomic analysis failed to detect calcium-dependent antibiotics. Interestingly, actinorhodin BGC (*act*) expression was repressed at 24 h under increased salinity and, then, subsequently activated at 48 h, but to a lesser degree (Fig. 6D). Indeed, the MS signals corresponding to actinorhodin were relatively smaller compared to those of coelimycin P1 and undecylprodigiosin (Fig. 1B and Fig. S5). The delayed and relatively lower activation of the *act* BGC could lead to reduced activation of actinorhodin production. The direct regulation of these four BGCs by PhoP is still debatable as various studies showed contradicting results (79). We analyzed the expression levels of the transcriptome data from the phosphate-limited conditions (76). In contrast to our data, the *cpk* BGC was repressed under the phosphate-limited condition, while the *red* BGC was not detectably affected (Fig. S12). The *act* BGC was more highly activated under the phosphate-limited condition, while the activation of the *cda* BGC was more limited. It is, therefore, less likely that phosphate depletion directly caused the activation of *red* and *cpk* expression, although it could still contribute to *act* and *cda* activation.

Unexpectedly, two siderophore BGCs, *cch* and *des*, were downregulated by increased salinity (Fig. 6E and F). The iron-dependent regulator, DmdR1, directly controls transcription of both BGCs together with the *desJGH* operon located outside of the *des* BGC (80). Of the latter three genes, *desG* and *desH* were downregulated, suggesting reduced production of siderophores and iron uptake (Fig. S13A). How increased salinity affects iron uptake and utilization is yet to be unveiled.

Biosynthesis of coelimycin, undecylprodigiosin, and actinorhodin, all of which are polyketides, requires malonyl-CoA as the biosynthetic substrate. Malonyl-CoA is synthesized from acetyl-CoA catalyzed by acetyl-CoA carboxylase. *S. coelicolor* encodes an essential acetyl-CoA carboxylase α subunit and a β subunit, AccA2 and AccB, respectively, which are required for fatty acid biosynthesis (81). Additionally, *S. coelicolor* encodes a second copy of an α subunit, AccA1, and a probable β subunit, CpkK (82). While the essential subunit genes were not significantly differentially expressed, these additional copies were substantially upregulated by increased salinity (Fig. S13B). Both *accA1* and *cpkK* are located within the *cpk* BGC. Although fatty acid biosynthesis competes with polyketide biosynthesis as both pathways use malonyl-CoA as the substrate, genes encoding fatty acid biosynthetic enzymes were not upregulated, suggesting enhancement of malonyl-CoA biosynthesis for polyketide production. Unlike the majority of the genes in the *cpk* BGC, AccA1 and CpkK together with CpkPα and CpkPβ are highly conserved among *Streptomyces* species (Fig. S14). Although the actual function of CpkPα and CpkPβ is still unknown, they are presumed to be involved in acetyl-CoA production (20). It is interesting to determine whether the orthologs of these conserved genes are highly expressed by increasing salinity to provide malonyl-CoA and enhance polyketide production in other *Streptomyces* species.

PKSs and NRPSs are activated by incorporation of a phosphopantetheinyl side chain of coenzyme A into their acyl carrier protein (ACP) or peptidyl carrier protein (PCP) domain, which is catalyzed by phosphopantetheinyl transferase (PPTase) (11). *S. coelicolor* encodes three PPTase genes, *acpS*, *redU,* and *SCO6673* (83). While AcpS is essential and activates fatty acid synthase and actinorhodin synthase ACPs, RedU and SCO6673 activate undecylprodigiosin synthase ACP and calcium-dependent antibiotic synthetase PCP. Both *redU* and *SCO6673* were highly expressed under increased salinity, while the *acpS* transcription level did not show any detectable change (Fig. S13C).

Secondary metabolism is often controlled by various transcription factors located outside the BGCs. One of them, AfsS, is a pleiotropic regulator that enhances *red* and *act* BGC expression, although AfsS target genes and how AfsS activates these BGCs are still unknown (84). *afsS* transcription is activated by AfsR, whose activity requires phosphorylation catalyzed by AfsK (85). Transcription of *afsS* was enhanced by salt supplementation, while the level of enhancement was lesser on the *afsR* transcription (Fig. S15). The *afsK* transcription was not detectably affected. AfsK activity is known to be controlled by autophosphorylation, which subsequently activates AfsR through phosphorylation. It is likely that increased salinity induces autophosphorylation of AfsK and phosphorylated AfsR activates *afsS* transcription. NsdA and NsdB negatively affect the production of various secondary metabolites, although the actual mechanism of regulation is yet to be known (86, 87). Transcription of both *nsdA* and *nsdB* was suppressed at 24 h under the increased salinity condition, which could additionally enhance secondary metabolite production.

In addition to differential expression of coding sequences (CDSs) of BGCs, we observed antisense transcription of hopene BGC (Fig. S15). Noticeably, the level of antisense transcription decreased under the increased salinity condition. Although the actual roles of antisense transcription vary, many of them are postulated to negatively control stability or translation of the complementary sense transcription (e.g., translation of the CDSs) (88, 89). Hopenes are known to reduce membrane permeability, possibly as a stress response (90). It was proposed that hopene diminishes water permeability of mycelia during morphological differentiation in *S. coelicolor* (91). It is plausible that hopene production is enhanced by increased salinity presumably to reduce the diffusion of water from cells.

We noticed activated expression of *SCO5746*, which encodes 3-amino-5-hydroxybenzoic acid (AHBA) synthase. (Fig. S13F). This enzyme catalyzes the last step of AHBA biosynthesis (92). AHBA is a precursor of various secondary metabolites several *Streptomyces* species produce, such as rifamycin and mitomycin (93). This gene in the *S. coelicolor* genome is not co-located with any genes likely involved in secondary metabolite biosynthesis and, interestingly, is located between a 5S rDNA gene and a tDNA gene. This gene was also highly expressed under low phosphate conditions, suggesting that accelerated phosphate depletion enhanced the expression of *SCO5746* (Fig. S12E). Thus far, no AHBA-derived secondary metabolite has been discovered from *S. coelicolor*. Further study of these growth conditions may reveal the role of AHBA in *S. coelicolor*.

Overall, the transcriptome data indicate that *S. coelicolor* activates the expression of genes involved in biosynthesis, precursor supply, posttranslational modification of PKS and NRPS, and regulation to enhance secondary metabolite production upon increased salinity.

#### Cell wall biosynthesis

Various genes known to be involved in cell wall biosynthesis were differentially expressed. These genes include the teichulosonic acid-cell wall biosynthetic gene cluster (*SCO4873–SCO4882*), which is controlled by PhoP (Fig. 5A) (94). Additionally, the first gene of the *cwg* operon, *cwgA*, and its divergently transcribed gene, *SCO6178*, are activated (Table S5). The latter gene encodes a glycoside hydrolase, which might modify peptidoglycan components. Both *cwg* and *SCO6178* are directly controlled by the cell envelope stress response sigma factor, σ^E^ (95). However, the *sigE* gene was not upregulated under the condition used in this study. Because SigE activity is known to be controlled at the transcriptional stage through conditional activation of *sigE* expression, it remains to be determined how transcription of these SigE target genes was activated under increased salinity (96). Additionally, *SCO1396*, encoding D-Ala-D-Ala dipeptidase, was also upregulated. Unlike the teichulosonic acid-cell wall biosynthetic genes, the *cwg* operon and *SCO1396* were not affected by phosphate limitation. Change in the cell wall property by salinity stress is a known phenomenon in other bacteria (97). Our data suggest that cell wall remodeling likely took place in response to increased salinity partially induced by phosphate limitation.

#### Transporters

In addition to the genes described above, several genes encoding ABC transporters were differentially expressed in response to salt supplementation (Fig. S16). While the substrates of many transporters are poorly known or predicted, there are some noticeable exceptions. The Pst transporter, which is activated by PhoP, is responsible for phosphate uptake upon phosphate limitation. Another transporter predicted to bind to phosphate, SCO2428, was also upregulated, although its involvement in phosphate limitation stress response was not previously mentioned. The transcriptome data from the phosphate limitation stress also showed upregulation of *SCO2428*, indicating induction of its expression by phosphate limitation stress. Another transporter, SCO5035, is predicted to transport lipoproteins. The glycine/betaine transporter genes, *SCO1620* and *SCO1621*, were actively expressed, as described above (Fig. 4B). In addition, a putative methionine transporter gene, *SCO1557*, was activated (Fig. S16). In contrast, the polyamine transporter genes (*SCO5667–SCO6570*) and dipeptide transporter genes (*SCO5476–SCO5480*) were partially downregulated, although the roles of these metabolites are not yet known.

In our transcriptomic and metabolomic analyses, a complex medium, ISP-2, was used. This medium, in addition to glucose, consists of yeast extract and malt extract, which contain various amino acids. Because of the known roles of small peptides in salinity stress response, it could be advantageous to activate various transport systems to uptake such protectants from the medium and to cope with the increased salinity (98). These observations suggest *S. coelicolor* changes the uptake rate of various compounds to adapt to increased salinity.

### Regulatory elements associated with salinity stress-induced genes

**Fig 7:**
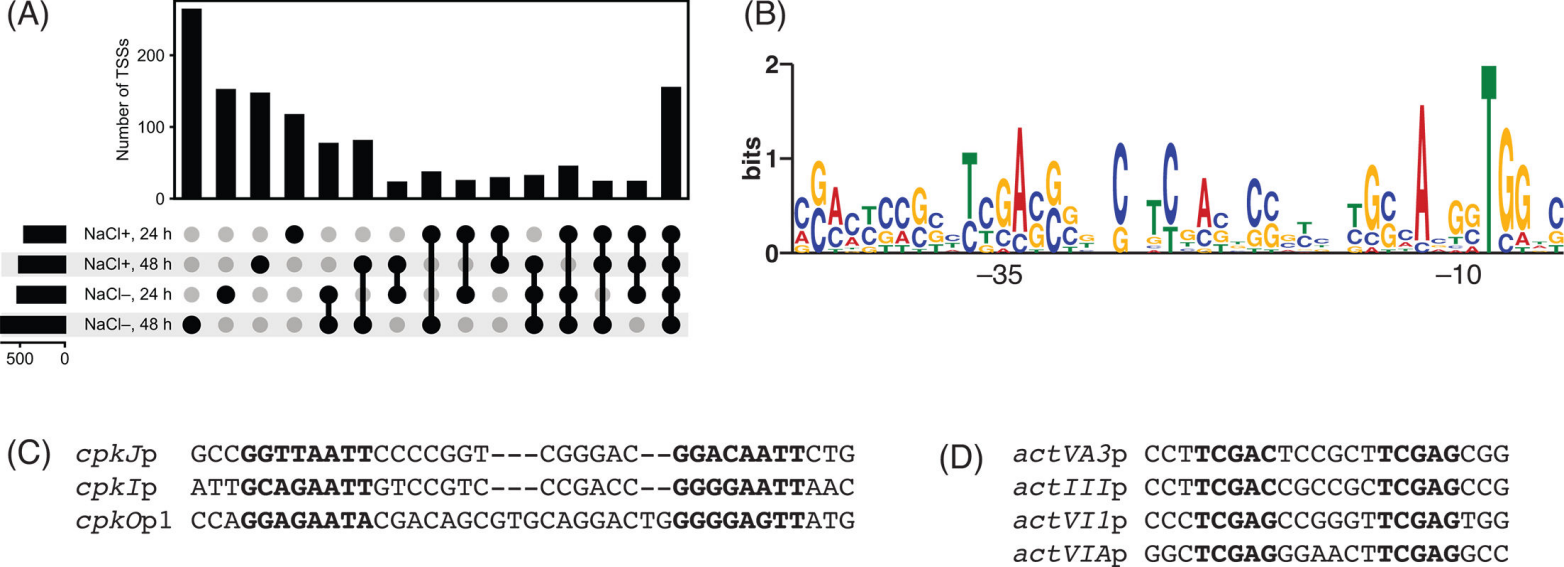
Transcriptional start sites (TSSs) identified by Cappable-seq. (**A**) Upset plot of TSSs detected under different conditions. (**B**) Consensus promoter motif of differentially expressed genes. (**C**) Alignment of three promoter sequences identified in the *cpk* BGC. (**D**) Alignment of four promoter sequences identified in the *act* BGC.

In order to better understand the mechanism of transcriptional regulation, we determined the TSSs by Cappable-seq (28). Since Cappable-seq requires a greater amount of RNA, we used RNA samples from each growth condition collected at 24 h and 48 h. We considered 5′ ends of sequencing reads as TSSs only if they align to the same position in all of 3 replicates at least under one of the growth conditions and time points. A total of 2,187 TSSs were identified by Cappable-seq (Table S6). Since many TSSs were identified under multiple conditions, the total number of individual or unique TSSs was 1,217. About half of the TSSs (595 TSSs) were detected under more than 1 condition, and 154 TSSs were detected under all of 4 conditions (Fig. 7A). We compared the TSSs identified in this study to the previous study of global TSS identification (99). A total of 555 TSSs were also identified in the previous study. This difference could be in part due to the different growth conditions and sequencing library preparation method used for TSS identification. Of 1,217 TSSs, 730 were located within 400 nt upstream from predicted genes, while 593 overlapped with genes either on the same strand (557 internal TSSs) or opposite strand (36 antisense TSSs). A total of 34 TSSs were not located within 400 nt of upstream from nor inside genes. Of 730 TSSs located upstream from genes, 101 TSSs were found upstream of differentially expressed genes, including 31 TSSs located upstream of genes upregulated under the increased salinity condition. We compared the promoter regions of TSSs located upstream from differentially expressed genes (Fig. 7B). Only 12 promoters possessed sequences resembling previously determined ones, although conservation of the –35 region was lower (99). This result suggests that many differentially expressed genes could be controlled by other regulatory proteins such as transcriptional activators and alternative sigma factors to enable conditional expression. Indeed, various transcription factor genes, particularly two-component regulatory systems, are differentially expressed (Fig. S17; Table S5). Of the two-component system genes differentially activated, *rapA1*/*A2* expression was induced at the early time point. RapA1 and RapA2 are a response regulator and a histidine kinase, respectively, known to positively control *act* and *cpk* BGC expression, although their target genes are unknown (100). The other substantially activated two-component regulatory system was *SCO2517* (response regulator) and *SCO2518* (histidine kinase). These two genes are located downstream of and presumably co-transcribed with *SCO2519*, encoding a membrane protein likely involved in sensing external stimuli to activate the histidine kinase. Other regulator genes likely to positively control transcription and induced in response to increased salinity are *wblH* (WhiB-family transcriptional activator) and *SCO4146* (sigma factor), although their functions remain to be determined. Overall, activation of these positive regulator genes likely induced various genes with diverse promoter sequences under the increased salinity condition.

Cappable-seq was able to identify the TSS of the antisense transcript within the *nuo* gene cluster (Fig. S9A). The previous study of genome-wide identification of PhoP-binding sites by ChIP-on-chip showed PhoP binding inside *nuoF* (101). Indeed, the promoter region of the antisense transcript originating inside *nuoF* harbors a sequence resembling the PHO box (Fig. S8B). As PhoP is also known to negatively control the transcription of some target genes (79) and phosphate limitation reduces the level of this antisense transcription (Fig. S9B) (76), PhoP is likely to directly repress the antisense transcription of *nuoF* and its surrounding genes to control the NADH dehydrogenase activity.

Many of the 31 TSSs located upstream of the gene upregulated under the increased salinity condition are inside BGCs including 7 TSSs in the *cpk* BGCs, 6 TSSs in the *act* BGC, 4 TSSs in the *cda* BGC, and 1 TSS in the *red* BGC. Importantly, some of these TSSs were detected even without salinity supplementation, suggesting low- or basal-level transcription in the absence of salinity change. Of the 7 TSSs detected in the *cpk* BGC, *scbR*p, *cpkO*p1, *cpkI*p, and *cpkK*p were identified in the previous study, while 3 promoters (*cpkD*p, *cpkO*p2, and *cpkJ*p) were not identified before (99). Comparison of these promoters revealed the presence of a direct repeat (GGNNAATT) in *cpkJ*p, *cpkI*p, and *cpkO*p1 (Fig. 7C). Although ScbR2 binding to the *cpkI* and *cpkJ* promoters was previously shown, its binding sites are different from this direct repeat (102). Instead, this consensus motif might be recognized by transcription factors activating the expression of this BGC such as CpkO (82). Similarly, 4 promoters in the *act* BGC contain a direct repeat (TCGAG), which resembles the known recognition motif of the SARP-family transcriptional activator (Fig. 7D) (103). These data enabled identification of DNA sequence motifs likely involved in regulation of secondary metabolite BGC expression induced by salt supplementation.

## DISCUSSION

**Fig 8:**
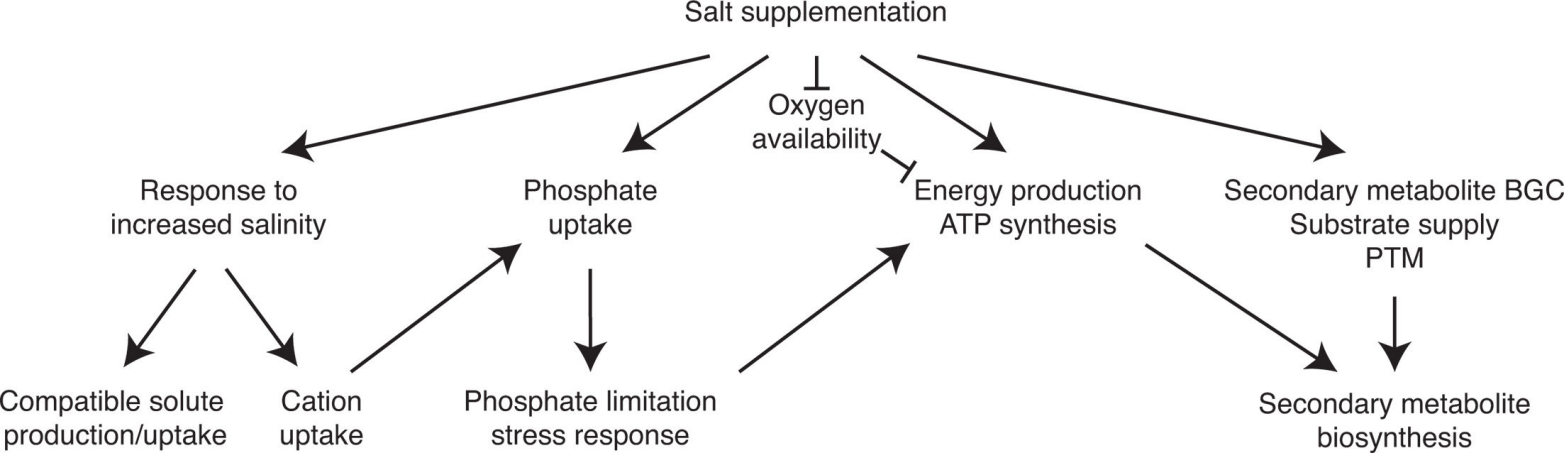
Model of the salinity stress response inducing secondary metabolite production.

Although it has long been known that various secondary metabolite biosynthetic pathways are activated by environmental stimuli, there is still little known about the physiological conditions that lead to such metabolic reprogramming and the broader genetic targets (104). In this study, we conducted a comprehensive analysis of transcriptomic and metabolomic responses to salinity change in *S. coelicolor* as this condition induced the most substantial change in the pigmented metabolite production. Our analyses revealed drastic changes that this condition induces in this biological system (Fig. 8). To cope with the increased salinity, various genes involved in cation uptake, compatible solute production, and possibly uptake of other compounds are activated. One of the transporters, KcsA, imports potassium or divalent cations using polyphosphate as a part of its cation-specific ion channel (68). We also observed accelerated depletion of phosphate from the culture medium and induced phosphate limitation stress response as a result. It is intriguing to suspect that the enhanced phosphate usage through the increased salinity could cause accelerated phosphate depletion from the media, presumably by the increased activity of KcsA and other proteins using phosphate. Consistently, no KcsA homolog is encoded in *S. griseofuscus*, *S. venezuelae,* and *S. rimosus*, where accelerated phosphate depletion was not detectably observed upon increased salinity (6). Nevertheless, the relationship between cation and phosphate uptake needs to be fully investigated. As a result of accelerated phosphate depletion, some of the known target genes of PhoP, phosphate limitation stress response regulator, were activated. These include the phosphate transporter genes, *pstSABC*, and the teichulosonic acid-cell wall biosynthetic genes, *SCO4873–SCO4882*. Teichulosonic acid-cell wall lacks phosphate, and activation of its biosynthetic genes together with other genes involved in cell wall biosynthesis suggests remodeling of the cell wall constituents. Importantly, there are several PhoP target genes whose expression level changed opposite to previous observations. For example, genes involved in oxidative phosphorylation were previously determined to be negatively controlled by PhoP (101). However, our data, together with those of another study, reveal activation of the NADH dehydrogenase genes in response to phosphate limitation, suggesting accelerated ATP production due in part to phosphate limitation (76). The remainder of the oxidative phosphorylation was activated presumably independent of the phosphate limitation response. Previous studies in mycobacteria showed a connection between oxygen limitation and oxidative phosphorylation (105). While the actual mechanism of this regulation is still to be elucidated, reduced oxygen solubility in the salt-supplemented medium could partially contribute to enhanced oxidative phosphorylation to maximize oxygen utilization. Other examples of the activated cellular processes are secondary metabolism, particularly the *cpk* and *red* BGCs. Although the exact mechanism of their activation is still unknown under the salt-supplemented condition, it is less likely that phosphate limitation is the major inducer of the expression of these BGCs because of different trends in the expression patterns between our and previous transcriptome data (76). Various regulatory genes known to positively or negatively affect BGC expression are also differentially expressed by increased salinity consistent with their known roles. In addition to enhanced BGC expression and ATP production, genes involved in activation of PKSs and NRPSs as well as the precursor supply for the biosynthesis are highly expressed. Overall, salt supplementation induces a salinity response and tailors the cellular metabolism toward secondary metabolite production, particularly polyketides and nonribosomal peptides.

How salt supplementation changes the expression of close to 1,000 genes is still largely unknown. Our transcriptome data show various regulatory genes differentially expressed. In order to understand the regulatory pathways controlled by these transcription factors, it is necessary to identify their target genes. Because many transcription factors directly control transcription of their own genes together with some neighboring genes, some target genes may be inferred from the transcription patterns if they are all co-expressed (106). For example, the transporter genes, *SCO5034* and *SCO5035*, are colocalized with *SCO5036*, encoding a PadR-like transcription factor. Since the *SCO5036* transcription is also activated under the salt-supplemented condition, it is likely that SCO5036 directly controls the transcription of *SCO5034*, *SCO5035,* and *SCO5036*. However, some transcription factors are global and directly control the transcription of a broad number of genes. Therefore, it is still necessary to find out the target genes of transcription factors that are important under this increased salt condition and how their activity is controlled.

Why are multiple secondary metabolites produced by increased salinity? Undecylprodigiosin, actinorhodin, and calcium-dependent antibiotics are known to exhibit antimicrobial activities (78, 107, 108). These antimicrobial compounds are likely produced to protect the producing organism from competing microbes or the host organism from pathogens under stress (1, 109). For example, actinorhodin is known to be a redox-active antibiotic (108). Because a higher salt concentration may reduce the amount of dissolved oxygen, *S. coelicolor* might produce actinorhodin to cause oxidative damages to other organisms under reduced environments. This regulatory mechanism could be further controlled by redox-sensing transcription factors, similar to those found in other bacteria including *Streptomyces* (110, 111). Although coelimycin A, the product of the CPK biosynthetic pathway, is known to exhibit antimicrobial activity, it is unstable and non-enzymatically converted into coelimycin P1 and P2 (112). Because coelimycin P1 and P2 do not exhibit the antimicrobial activity coelimycin A possesses, it is less likely that the CPK biosynthetic pathway was activated to produce an effective “chemical weapon” (20). The *cpk* BGC encodes transcriptional repressors, ScbR and ScbR2, which directly repress the expression of the *cpk* BGC at an earlier and later time point, respectively (113). Interestingly, ScbR2 positively controls the expression of the *red*, *act,* and *cda* BGCs (102). While the ScbR activity is controlled by γ-butyrolactones (GBLs), SCB-1, SCB-2, and SCB-3, ScbR2 is a pseudo-GBL receptor binding to some secondary metabolites including undecylprodigiosin and actinorhodin (114). This GBL receptor-mediated regulation has been considered *Streptomyces* quorum-sensing mechanisms, enabling secondary metabolite production in a population-dependent manner (113). Indeed, accumulation of these SCBs was enhanced by salt supplementation, leading to *cpk* BGC activation. However, the existence of the GBL-independent regulatory mechanisms by the pseudo-GBL receptor and activation of SCB production by salt supplementation, where the biomass yield did not drastically change, suggest an additional layer of regulation. It is plausible that the *cpk* BGC was activated by a yet-to-be-discovered regulatory pathway, presumably by enhancing the ScbA activity, to produce ScbR2, which then activated the production of other secondary metabolites that serve as chemical weapons. These secondary metabolites, such as undecylprodigiosin and actinorhodin, further activate their BGCs via ScbR2 binding, allowing a feedforward loop of regulation. Alternatively, one of the other BGCs, such as *red* BGC, could at first be partially activated, which subsequently activates *cpk* and other BGCs via ScbR2. Further investigation is still needed to understand how increased salinity induces the *cpk* BGC expression and other BGCs and which BGC is the first one to be activated.

Although the response to increased salinity has been studied in this bacterium in the past, there are some differences between the prior observations and our data. For example, a prior study on supplementation of ~400 mM sodium chloride showed elevated undecylprodigiosin production while actinorhodin production was suppressed (43). Our data showed activation of the *act* BGC, although it was delayed compared to the timing of the *red* BGC activation. The difference could be due to the different concentrations of sodium chloride supplemented or the medium used. Our study also shows accumulation of other metabolites that the previous study did not measure, particularly coelimycin P1. There are some regulatory genes that are known to be induced under the high-salinity condition, including sigma factors such as SigB and two-component regulatory systems such as OsaABC (115, 116). The prior studies showed that transcription of these regulatory genes is transiently activated following exposure to increased salinity. Although our transcriptome data include 30 min after exposure to increased salinity, differential expression of these genes was not observed. This discrepancy could partly be due to the different and relatively lower salt concentration used in this study. A prior study of screening for finding SigB target genes found 16 genes likely controlled by SigB (117). However, none of these genes were substantially activated by salt supplementation, supporting potentially limited involvement of SigB in response to increased salinity studied here. Since the target genes of OsaB (response regulator) are unknown, the SigB regulon is still to be more fully elucidated, and the involvement of these regulatory proteins in enhanced secondary metabolism has not been examined yet, it would be interesting to determine how these regulatory proteins contribute to this response and whether they also directly control or affect the cellular metabolism, resulting in enhanced secondary metabolite production (115, 116, 118).

Our transcriptome data also showed that many genes that are differentially expressed are conserved in a relatively small number of species. One of the potassium transporter genes upregulated, *kcsA*, is conserved in only 3% of the *Streptomyces* genomes analyzed, while the other upregulated potassium transporter gene, *SCO3602*, is present in 71% of the *Streptomyces* genomes. Genes involved in compatible solute production such as ectoine synthase are more highly conserved (85%–95%) (6). These observations suggest that *Streptomyces* species primarily increase the potassium or other cation and solute concentrations via conserved mechanisms, while the species- or strain-specific pathway further accelerates accumulation of these molecules. Additionally, the malonyl-CoA biosynthetic genes induced by increased salinity are highly conserved, although polyketide biosynthetic genes are often far less conserved (6). Because malonyl-CoA is one of the most common precursors for polyketide biosynthesis, various *Streptomyces* species may accelerate the production of diverse polyketides upon salt supplementation, presumably to defend themselves or their hosts. It would be interesting to investigate how other *Streptomyces* species respond to salt supplementation, including whether similar metabolic reprogramming takes place for production of secondary metabolites and how common this response mechanism is.

Past studies in other bacteria, particularly *E. coli* and *B. subtilis*, have revealed the molecular mechanisms of how these bacteria cope with increased salinity including compatible solute and potassium uptake (119). This includes the activation of various transporters at transcriptional and posttranslational levels. In *B. subtilis*, a change in the lipid composition of the cell envelope is known, which is accompanied by the differential expression of genes involved in fatty acid biosynthesis and degradation (120, 121). Additionally, involvement of a peptidoglycan hydrolase in salinity stress response was shown, suggesting cell wall modification under this stress condition (122). Compared to those well-studied bacteria, knowledge on this response mechanism in *Streptomyces* species was fragmented. Members of the genus *Streptomyces* are known for their prowess to produce diverse secondary metabolites. This study reveals how this biological system adapts to the high-salinity condition and reprograms the cellular metabolism. Similar to other bacteria, *S. coelicolor* accumulates cations and compatible solutes including ectoine in response to increased salinity. However, unlike what is known in many other bacteria, this response mechanism triggers phosphate utilization and phosphate limitation stress response as a result. The combination of these two signals tailors the cellular metabolism toward the production of various secondary metabolites including those for increased substrate supply, induced expression of biosynthetic genes, and enhanced posttranslational modification activity. This metabolic reprogramming is, at least, partly controlled by yet-to-be-discovered regulatory pathways because of various differentially expressed regulatory genes and diversity of salinity-induced promoter sequences. Future studies on characterizing these regulatory genes and elements will unveil the detailed molecular mechanism controlling this physiological change that enables enhanced secondary metabolism and the roles that the secondary metabolites play under this environment in *Streptomyces* species.

## Data Availability

The whole transcriptome and Cappable-seq data files are available at the National Center for Biotechnology Information under accession number PRJNA1315997. The accession numbers of individual files are listed in Table S7. The metabolome data files are available at MassIVE under accession number MSV000098817.

## SUPPLEMENTAL MATERIAL

Supplemental Figures msystems.01718-25-s0001.pdf Figures S1 to S18.

Supplemental Tables msystems.01718-25-s0002.xlsx Tables S1 to S7.

ASM does not own the copyrights to Supplemental Material that may be linked to, or accessed through, an article. The authors have granted ASM a non-exclusive, world-wide license to publish the Supplemental Material files. Please contact the corresponding author directly for reuse.
