# Supplementary material for: Salt supplementation-induced metabolic reprogramming in Streptomyces coelicolor
Source: mSystems. 2026 Mar 3;11(3):e01718-25. doi: 10.1128/msystems.01718-25 (PMC13011467; doi:10.1128/msystems.01718-25)
Supplement: Supplemental Figures — Figures S1 to S18. [file msystems.01718-25-s0001.pdf]

Supplementary Information

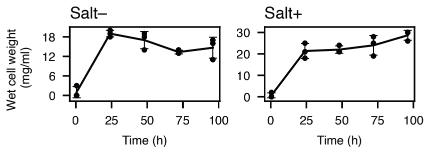

Supplementary Figure 1. Growth curves of *S. coelicolor* cultivated with or without salt supplementation. Error bars are standard deviation from three biological replicates.

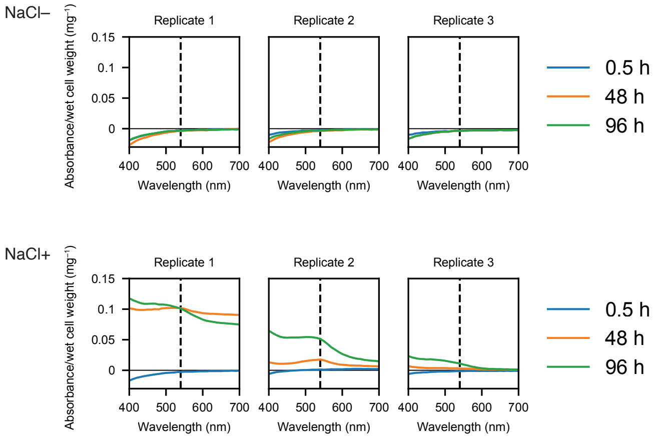

Supplementary Figure 2. Absorption spectra of the culture supernatants without (upper panels) or with (bottom panels) sodium chloride supplementation. The dashed vertical lines indicate 540 nm.

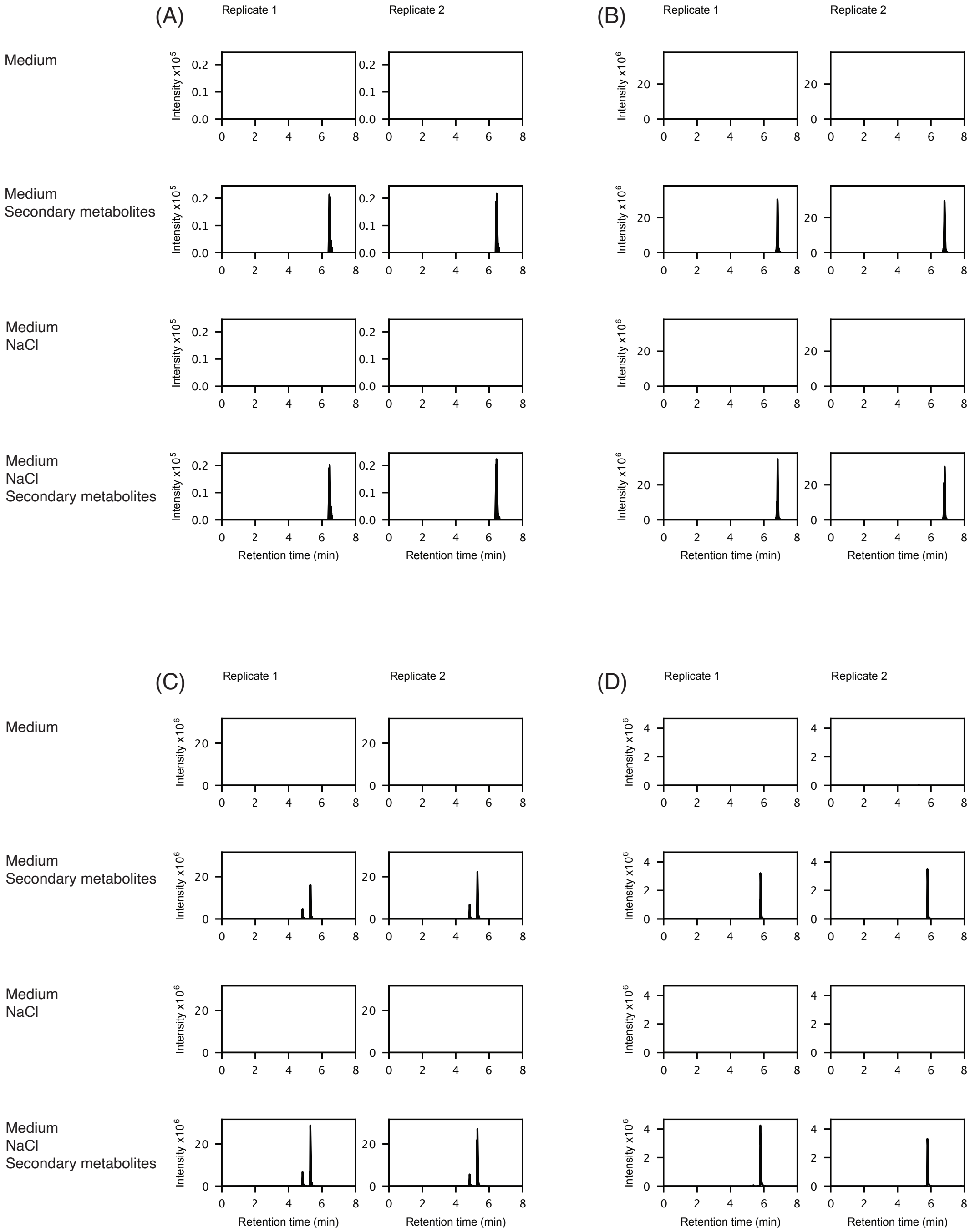
(A)
(B)
(C)
(D)
Replicate 1
Replicate 2
Medium
Medium
Secondary metabolites
Medium
NaCl
Medium
NaCl
Secondary metabolites
Intensity x10^5
Intensity x10^6
Retention time (min)

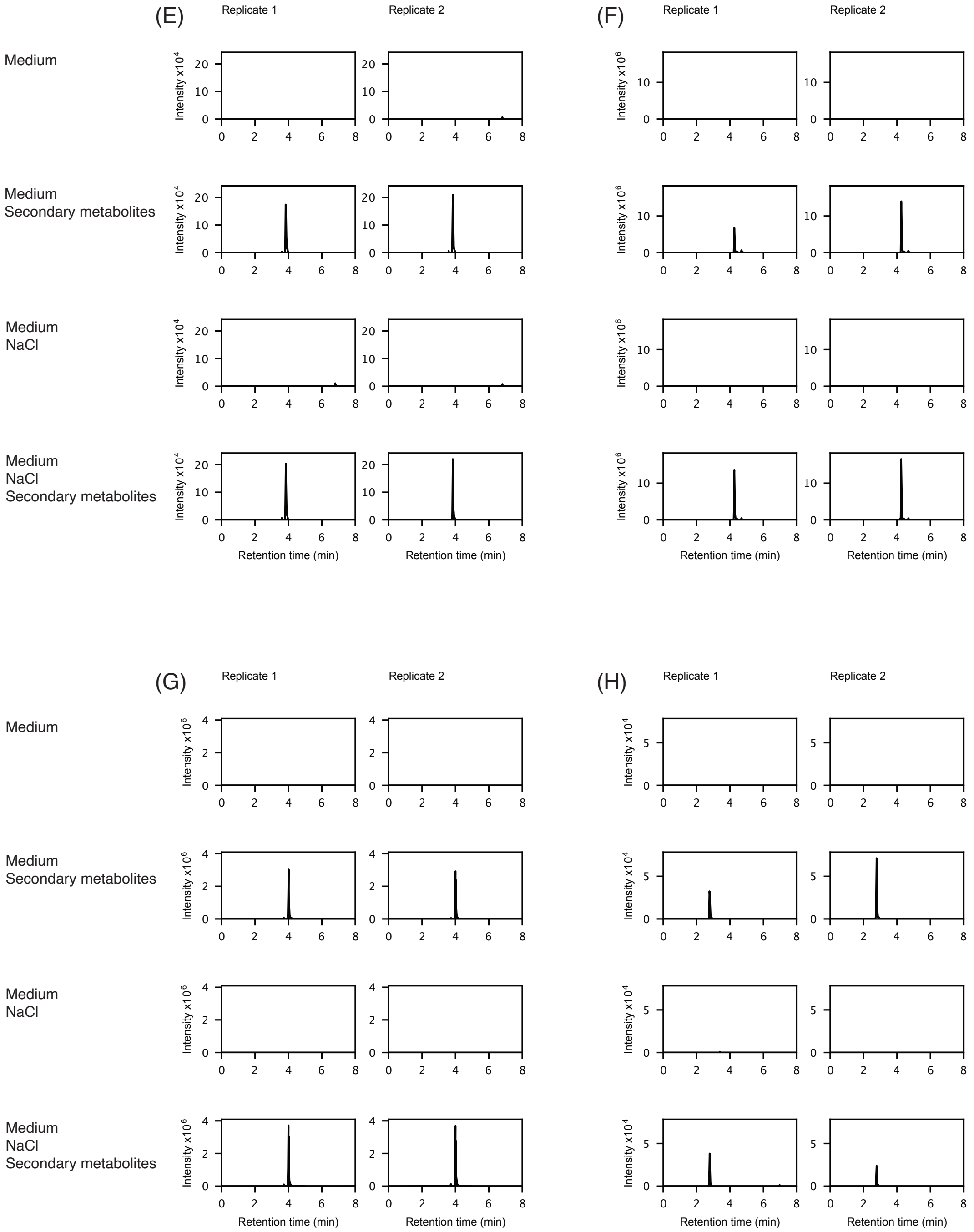

Supplementary Figure 3. Comparison of extraction efficiency with and without salt supplementation. The ISP-2 medium supplemented with or without 100 mM sodium chloride was spiked-in with 10 known secondary metabolites (1 µg/ml each) and extracted by ethyl acetate. All the extraction experiments were repeated twice. Note that the chromatograms of ivermectin and apramycin are omitted due to their poor extraction or ionization. (A)

Chromatograms for the *m*/*z* value of 392.271 ± 10 ppm in the negative ionisation mode, corresponding to undecylprodigiosin. (B) Chromatograms for the *m*/*z* value of 1255.64 ± 10 ppm in the positive ionisation mode, corresponding to actinomycin D. (C) Chromatograms for the *m*/*z* value of 823.41 ± 10 ppm in the positive ionisation mode, corresponding to rifampicin. (D) Chromatograms for the *m*/*z* value of 1664.50 ± 10 ppm in the positive ionisation mode, corresponding to thiostrepton. (E) Chromatograms for the *m*/*z* value of 323.02 ± 10 ppm in the positive ionisation mode, corresponding to chloramphenicol. (F) Chromatograms for the *m*/*z* value of 734.49 ± 10 ppm in the positive ionisation mode, corresponding to erythromycin. (G) Chromatograms for the *m*/*z* value of 425.14± 10 ppm in the negative ionisation mode, corresponding to anhydrotetracycline. (H) Chromatograms for the *m*/*z* value of 405.21 ± 10 ppm in the negative ionisation mode, corresponding to lincomycin.

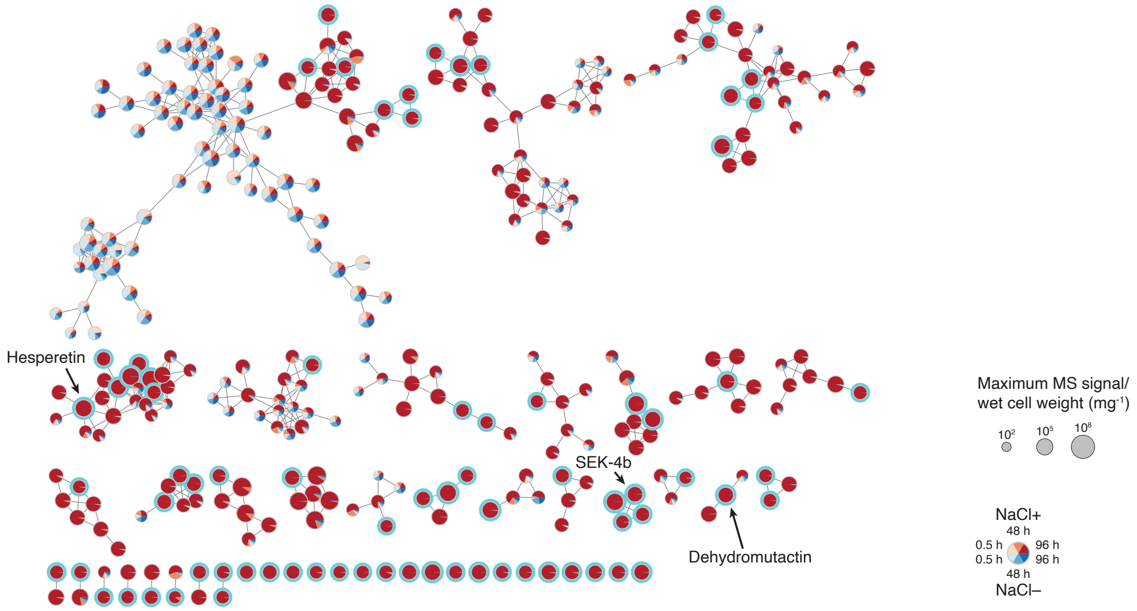

Supplementary Figure 4. Molecular network analysis of the metabolites detected in the negative ionisation mode. The nodes with a cyan outer circle indicate metabolites significantly differentially produced or depleted between two salinity conditions. Only clusters containing at least one metabolite significantly produced or depleted are shown. The node size indicates the maximum MS signal quantity per wet cell weight.

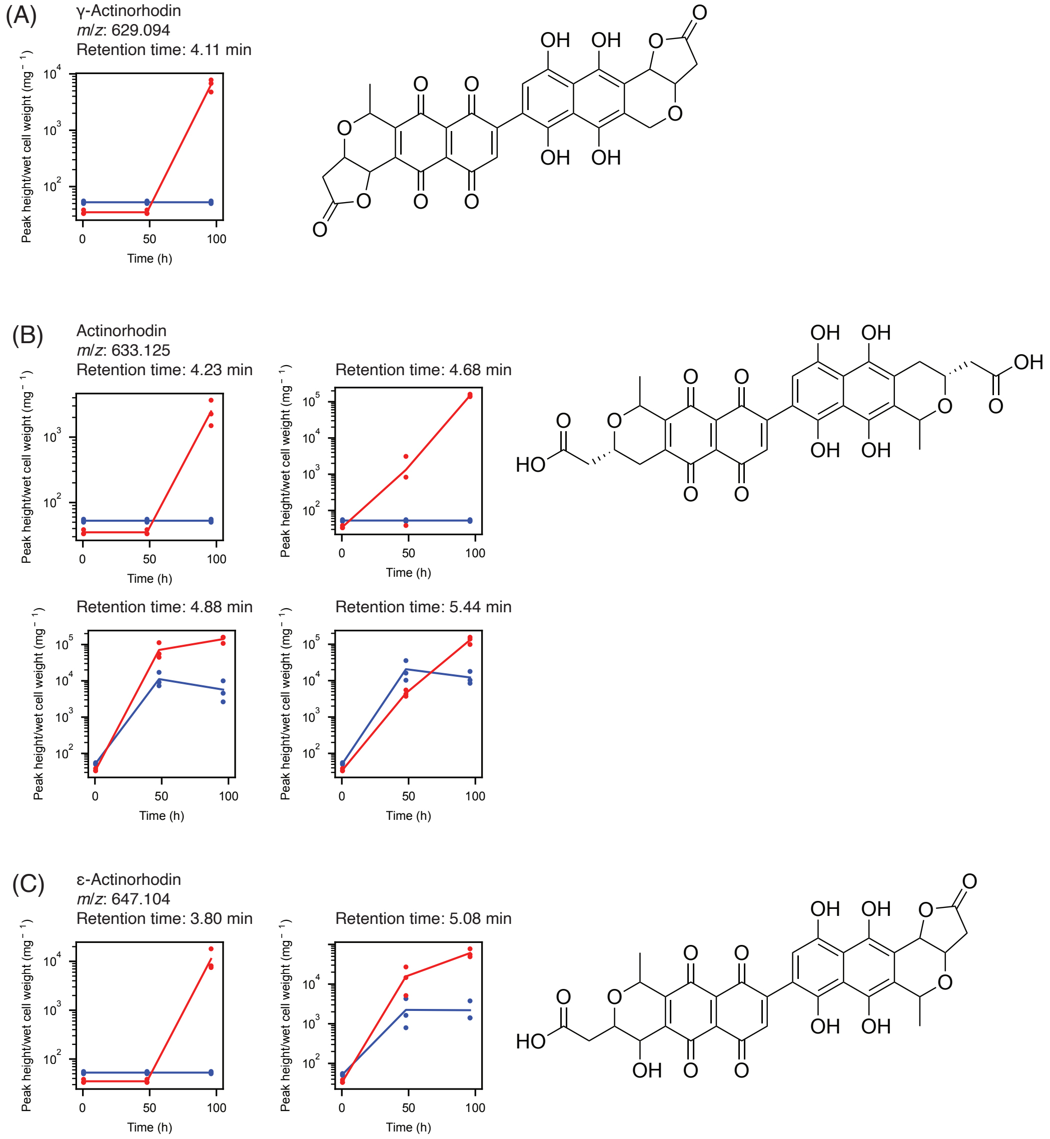

Supplementary Figure 5. Quantities of γ-actinorhodin (A), actinorhodin (B), and ε-actinorhodin (C) detected in the positive ionisation mode. Lines connect average values from three independent biological experiments. Values are normalised by wet cell weight. Red line and dots: high salinity condition. Blue line and dots: no additional salinity condition.

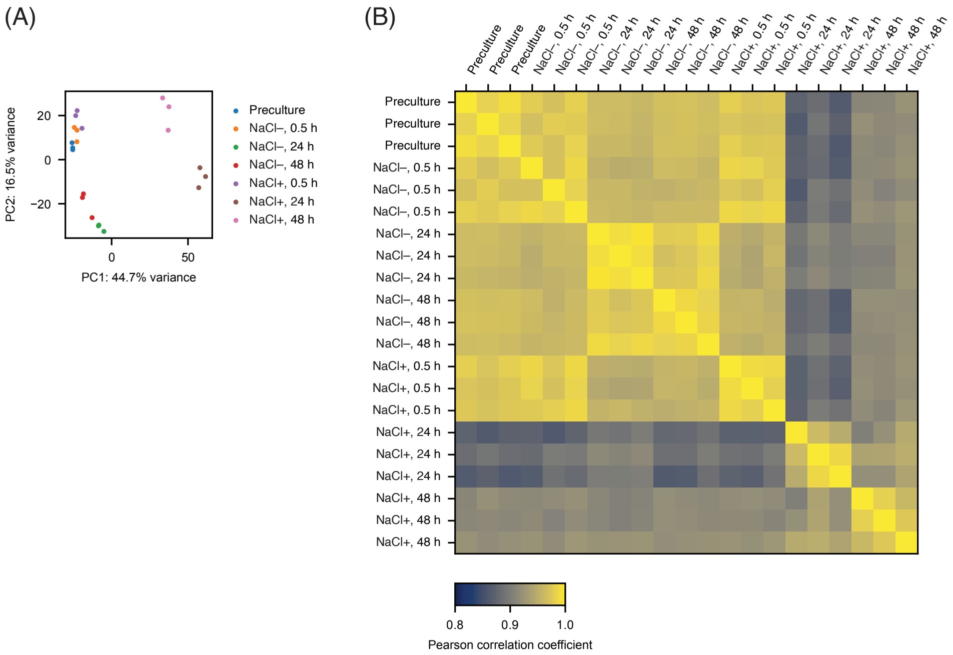

Supplementary Figure 6. (A) Principal component analysis of the sequence fragment count per gene between sequencing libraries. The first two components are shown. (B) Pearson correlation of the sequence fragment count per gene between sequencing libraries.

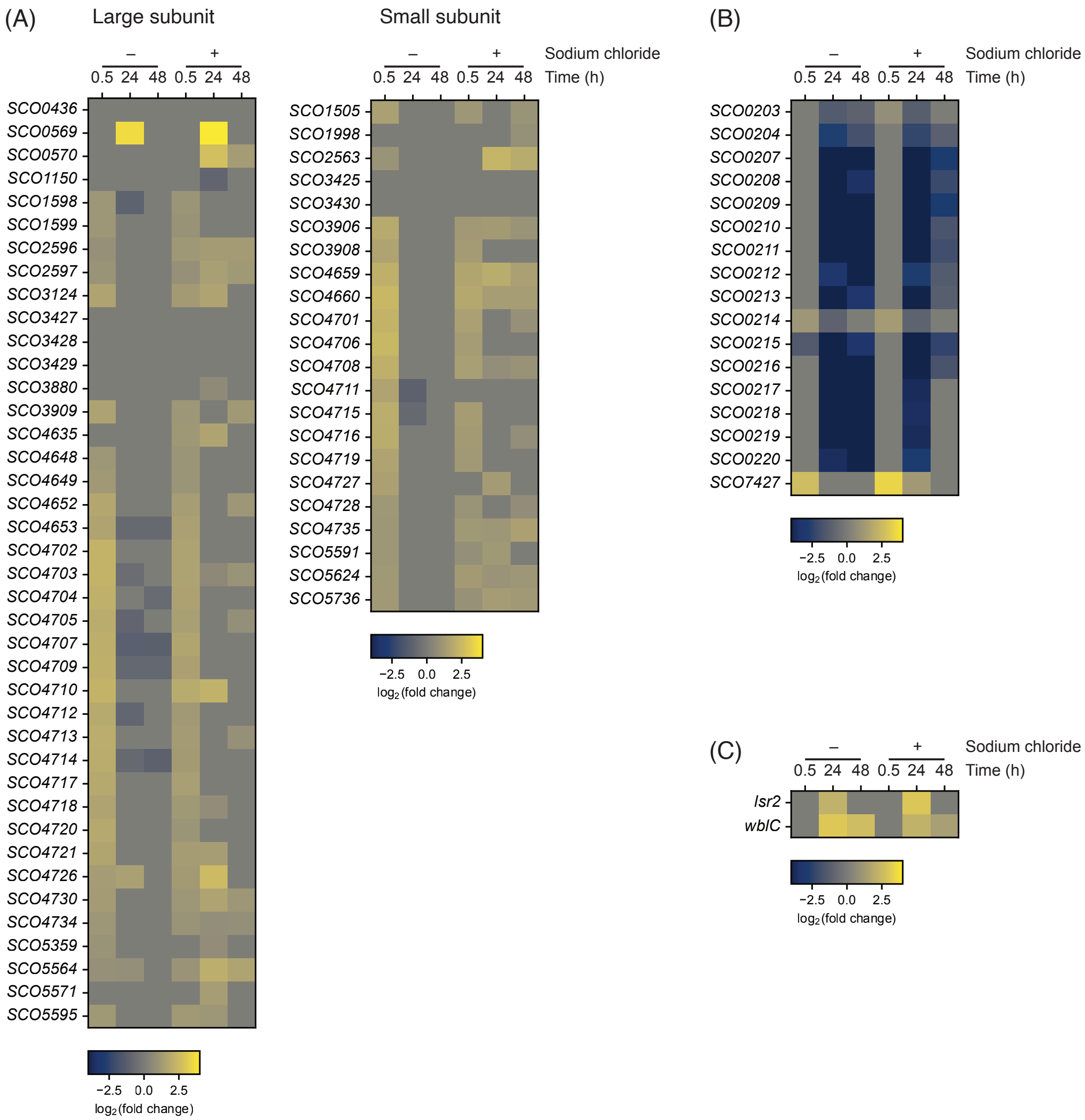

Supplementary Figure 7. Heatmap of genes (A) encoding ribosomal proteins, (B) involved in nitrate and nitric oxide reduction, and (C) encoding transcription factors mentioned in the main text. The colour-coding indicates log_2-transformed fold change relative to the reference condition.

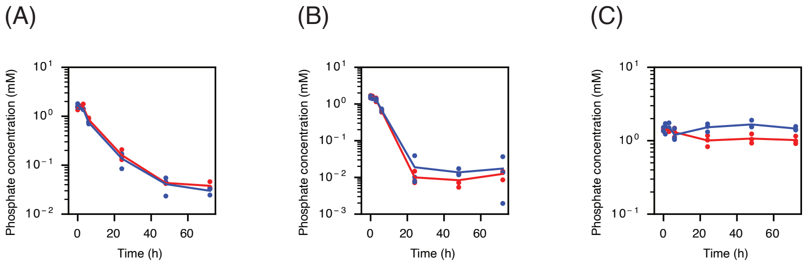

Supplementary Figure 8. Phosphate concentrations in the *S. griseofuscus* (A), *S. venezuelae* (B) and *S. rimosus* (C) culture media. Red line and dots: Salt-supplemented condition. Blue line and dots: no additional salinity condition. Lines connect average values from three independent biological experiments.

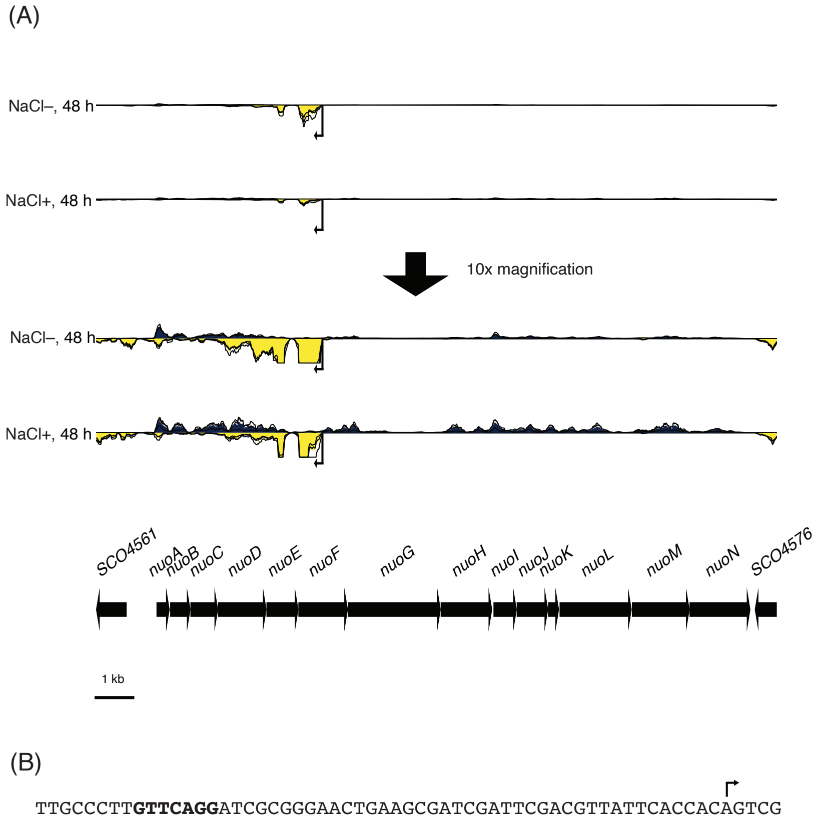

Supplementary Figure 9. (A) Transcription profile of the *nuo* locus at 48 hours. In blue and yellow are normalised average read counts on the sense (left to right) and antisense (right to left) strands, respectively. Bent arrows indicate the transcription start sites determined by Cappable-seq. (B) The promoter region of the antisense transcript. Bent arrow indicates the transcription start site and the bold letters indicate the PHO box.

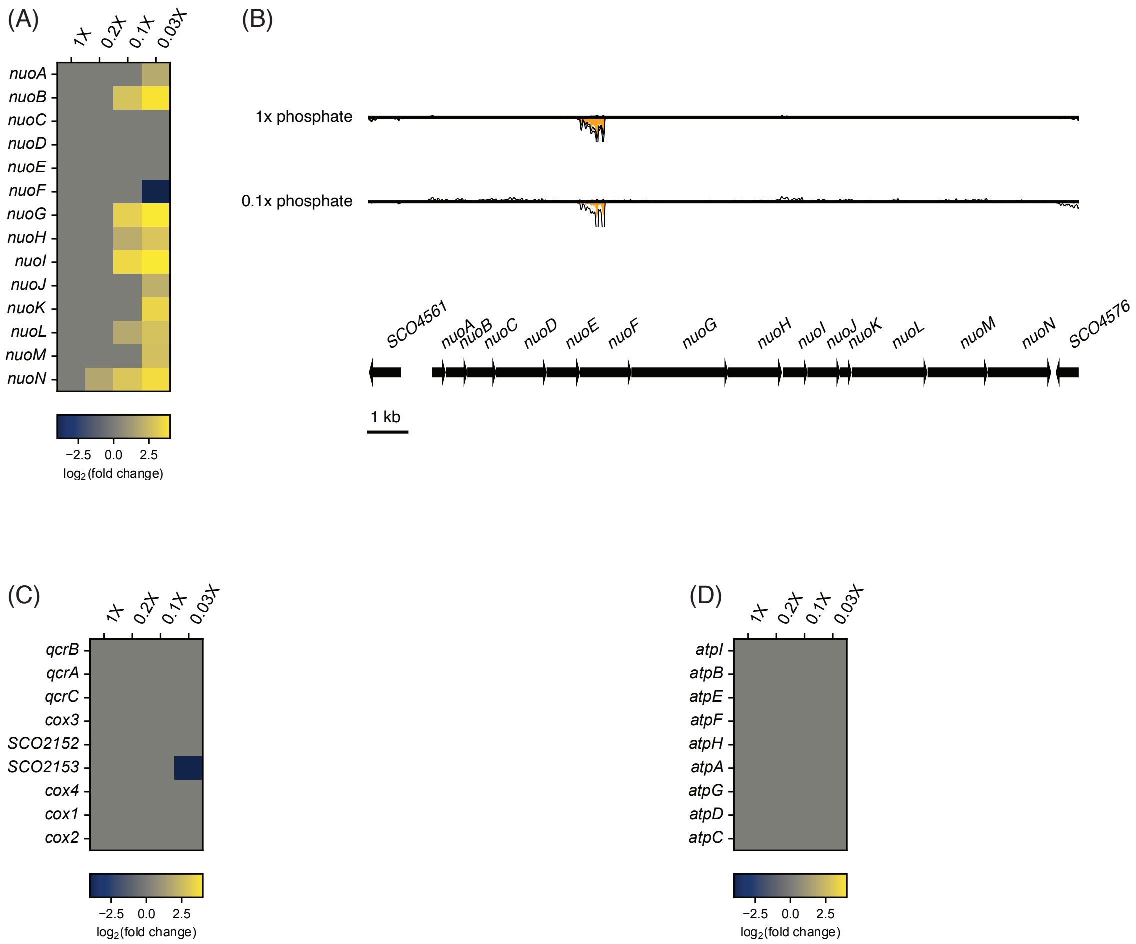

Supplementary Figure 10. (A) Heatmap of the relative expression levels of the *nuo* genes with varying phosphate availabilities compared to the 2x phosphate condition. (B) Transcription profile of the *nuo* locus with the 1x and 0.1 phosphate conditions. In blue and yellow are normalised average read counts on the sense (left to right) and antisense (right to left) strands, respectively. (C) Heatmap of the relative expression levels of the *cox* and *qcr* genes with varying phosphate availabilities compared to the 2x phosphate condition. (D) Heatmap of the relative expression levels of the *atp* genes with varying phosphate availabilities compared to the 2x phosphate condition.

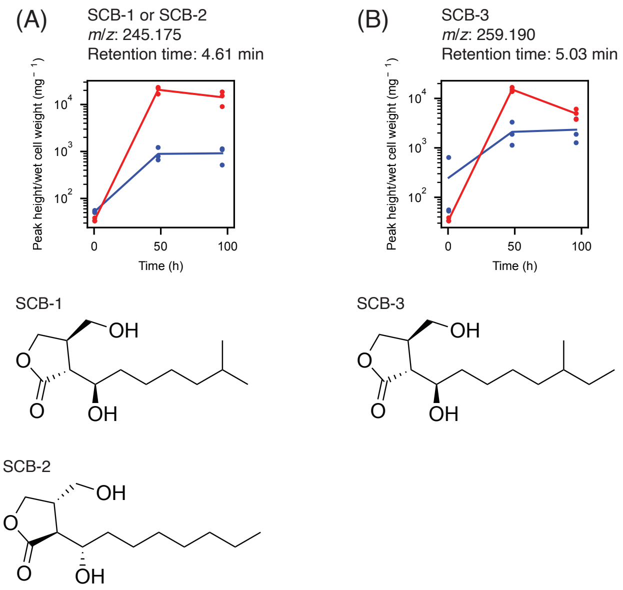

Supplementary Figure 11. Quantities of SCB-1 or SCB-2 (A), and SCB-3 (B) detected in the positive ionisation mode. Lines connect average values from three independent biological experiments. Values are normalised by wet cell weight. Red line and dots: high salinity condition. Blue line and dots: no additional salinity condition.

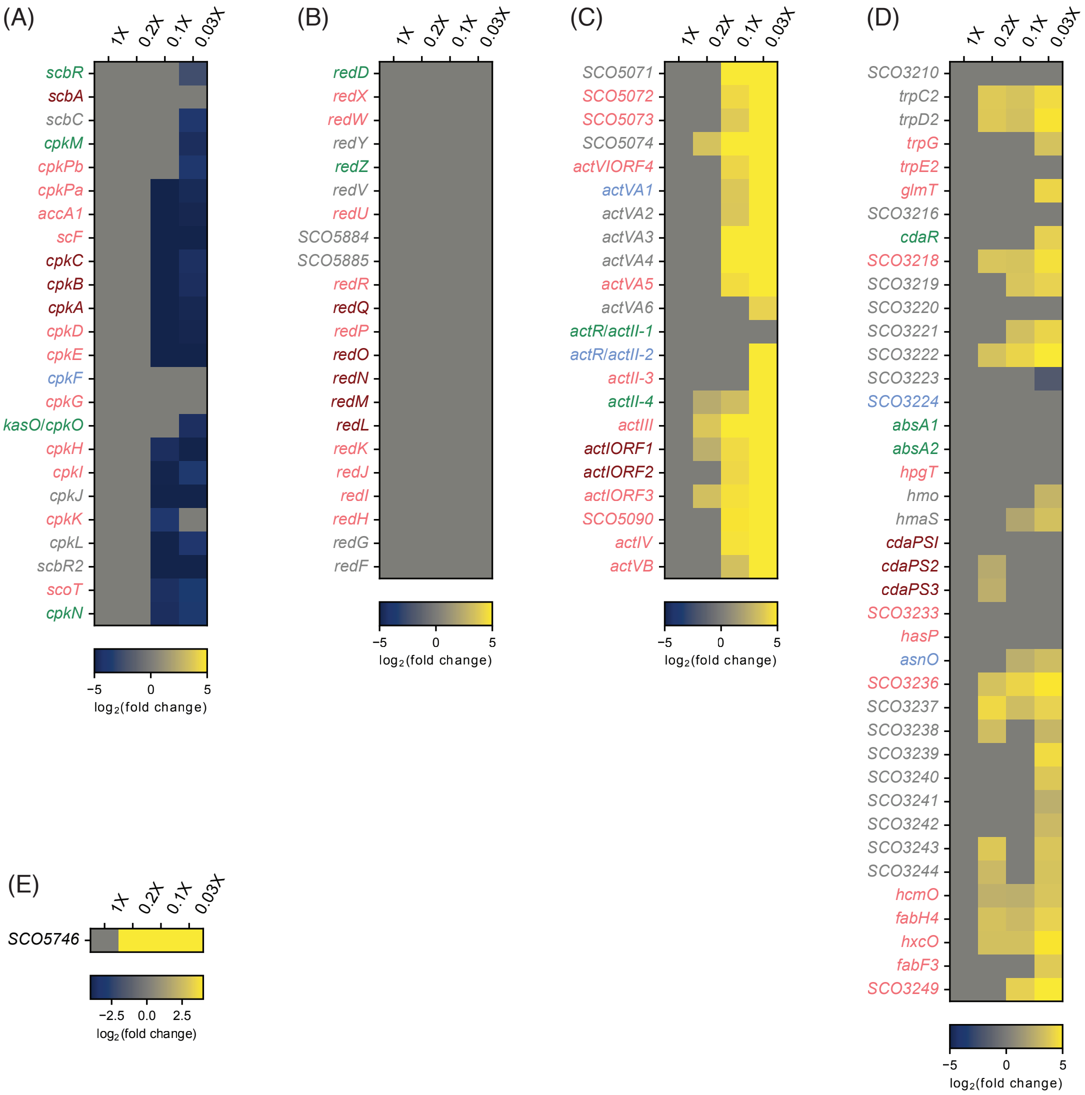

Supplementary Figure 12. Heatmap of the relative expression levels of the *cpk* BGC (A), the *red* BGC (B), the *act* BGC (C), the *cda* BGC (D) and the AHBA synthase gene (E) with varying phosphate availabilities compared to the 2x phosphate condition. The colour-codes of the genes indicate the functions (dark red: core biosynthetic genes, light red: additional biosynthetic gene, light blue: transport-related gene, green: regulatory gene, grey: other gene).

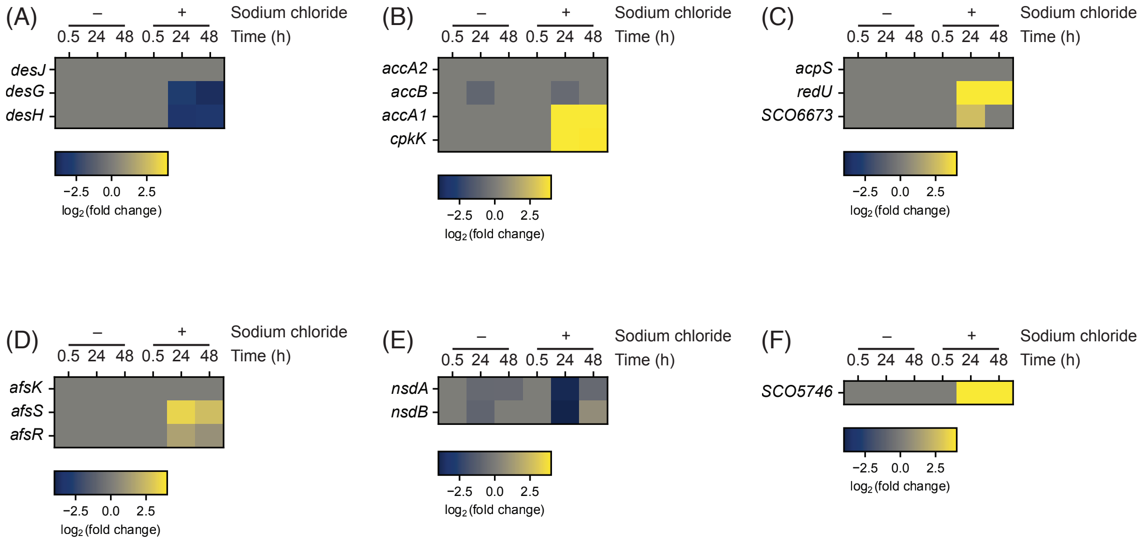

Supplementary Figure 13. Heatmap of (A) the *desJGH* genes, (B) the acetyl-CoA carboxylase genes, (C) the PPTase genes, (D) the *afs* genes, (E) the *nsd* genes, and (F) the AHBA synthase gene. The colour-coding indicates $\log_2$-transformed fold change relative to the reference condition.

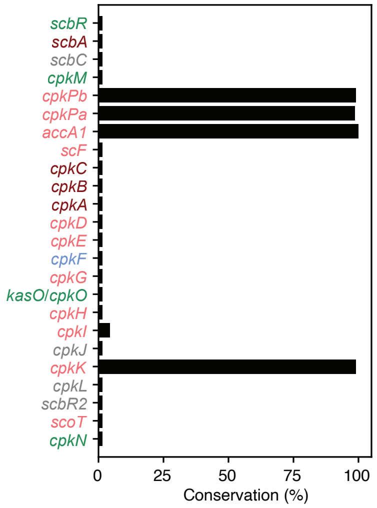

Supplementary Figure 14. Conservation of the *cpk* genes among 205 *Streptomyces* genomes. The colour-codes of the genes indicate the functions (dark red: core biosynthetic genes, light red: additional biosynthetic gene, light blue: transport-related gene, green: regulatory gene, grey: other gene).

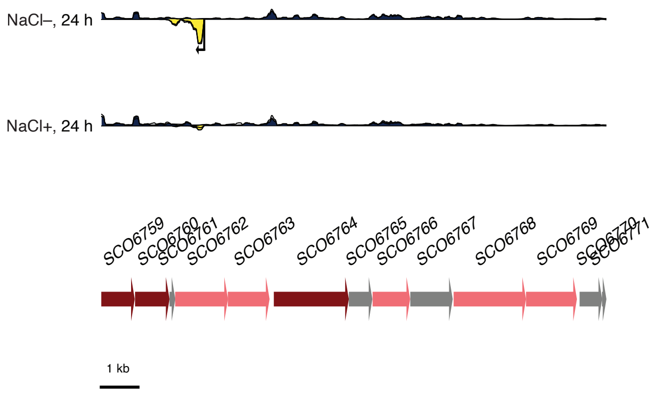

Supplementary Figure 15. Transcription profile of the hopene BGC at 24 hours. In blue and yellow are normalised average read counts on the sense (left to right) and antisense (right to left) strands, respectively. Bent arrows indicate the transcription start sites determined by Cappable-seq. The colour-codes of the arrows indicate the functions (dark red: core biosynthetic genes, light red: additional biosynthetic gene, light blue: transport-related gene, green: regulatory gene, grey: other gene).

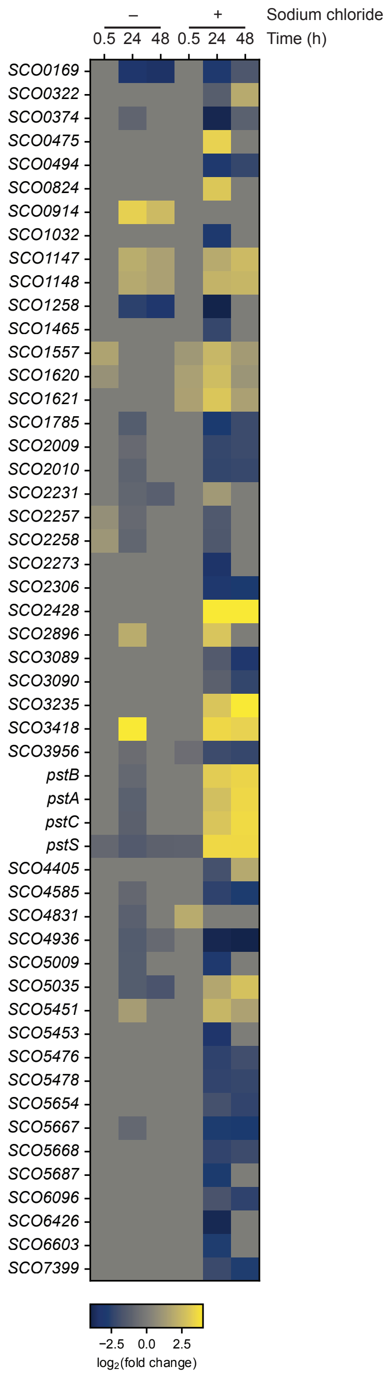

Supplementary Figure 16. Heatmap of the differentially expressed ABC transporter genes. The colour-coding indicates log_2-transformed fold change relative to the reference condition.

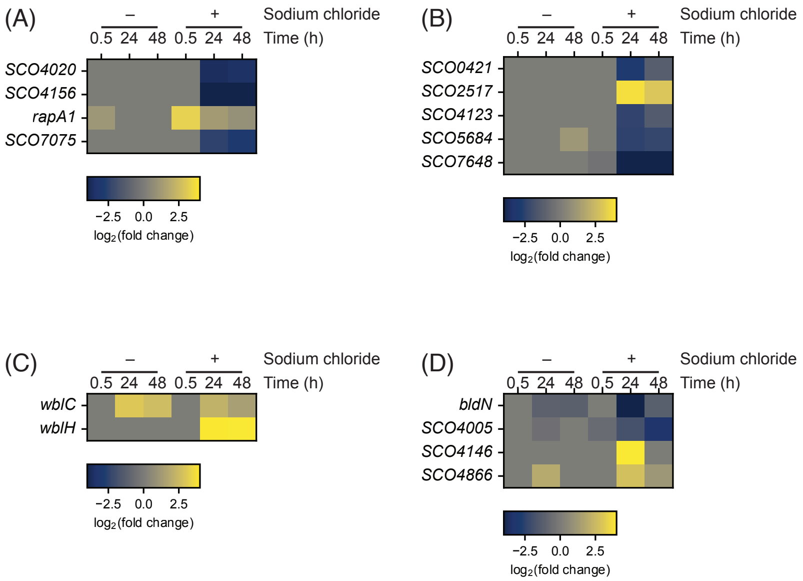

Supplementary Figure 17. Heatmap of the differentially expressed (A) two-component response regulator genes (no specific family), (B) LuxR-family two-component response regulator genes, (C) WhiB-family transcriptional activator genes, and (D) sigma factor genes. The colour-coding indicates log_2-transformed fold change relative to the reference condition.

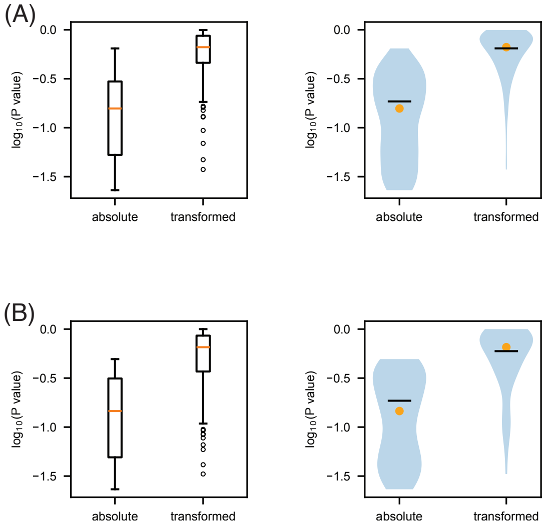

Supplementary Figure 18. Levene Test of the LC-MS features between six sample groups (three time points ± sodium chloride supplementation). Box plot (left) and violin plot (right) representation of the P values calculated by Levene Test using the absolute and log_{10}-transformed LC-MS signals detected in the positive (A) and negative (B) ionisation mode are shown. On the box plots, the orange horizontal lines indicate median values. On the violin plots, the black horizontal lines and orange dots indicate mean and median values, respectively.
